# What are the toxicity thresholds of chemical pollutants for tropical reef-building corals? A systematic review

**DOI:** 10.1186/s13750-023-00298-y

**Published:** 2023-03-19

**Authors:** Dakis-Yaoba Ouédraogo, Hugo Mell, Olivier Perceval, Karen Burga, Isabelle Domart-Coulon, Laetitia Hédouin, Mathilde Delaunay, Mireille M. M. Guillaume, Magalie Castelin, Christophe Calvayrac, Odile Kerkhof, Romain Sordello, Yorick Reyjol, Christine Ferrier-Pagès

**Affiliations:** 1 Direction de L’Expertise, Muséum National d’Histoire Naturelle (MNHN), 75005 Paris, France; 2 UMS Patrimoine Naturel (PatriNat), OFB-MNHN-CNRS, 75005 Paris, France; 3 Office Français de la Biodiversité (OFB), 94300 Vincennes, France; 4 French Agency for Food, Environmental and Occupational Health & Safety (ANSES), 94701 Maisons-Alfort Cedex, France; 5 Laboratoire Molécules de Communication et Adaptation des Microorganismes (MCAM), UMR 7245, CNRS-Muséum National d’Histoire Naturelle (MNHN), 75005 Paris, France; 6 Laboratoire d’Excellence CORAIL, 66860 Perpignan, France; 7 USR 3278 CRIOBE, PSL Université Paris : EPHE-UPVD-CNRS, 98729 Papetoai, Mo’orea, French Polynesia; 8 Laboratoire de Biologie Des Organismes et Ecosystèmes Aquatiques (BOrEA), Muséum National d’Histoire Naturelle-CNRS – SorbonneU – IRD – UCN - UA EcoFunc - Aviv, 75005 Paris, France; 9 Institut de Systématique, Evolution, Biodiversité (ISYEB), Muséum National d’Histoire Naturelle - CNRS - Sorbonne Université - EPHE - Université des Antilles, 75005 Paris, France; 10 Biocapteurs Analyses Environnement, University of Perpignan via Domitia, 66000 Perpignan, France; 11 Laboratoire de Biodiversité et Biotechnologies Microbiennes (LBBM), Sorbonne Universités - CNRS, 66650 Banyuls Sur Mer, France; 12 Equipe Ecophysiologie Corallienne, Centre Scientifique de Monaco, MC-98000 Monaco, Monaco

**Keywords:** Contamination, Hazard assessment, Hermatypic, No observed effect concentration, Pollution, Scleractinian, Toxicity endpoints

## Abstract

**Background:**

Tropical coral reefs cover only ca. 0.1% of the Earth’s surface but harbour exceptional marine biodiversity and provide vital ecosystem services to millions of people living nearby. They are currently threatened by global (e.g. climate change) and local (e.g. chemical pollution) stressors that interact in multiple ways. While global stressors cannot be mitigated by local actions alone, local stressors can be reduced through ecosystem management. Here, we aimed to systematically review experimental studies assessing the toxicity of chemical pollutants to tropical reef-building corals to generate accessible and usable knowledge and data that can be used to calculate measurement endpoints in ecological risk assessment. From the quantitative estimates of effects, we determined toxicity thresholds as the highest exposures tested at which no statistically significant adverse effects were observed, and we compared them to regulatory predicted no effect concentrations for the protection of marine organisms, to assess whether these reference values are indeed protective of corals.

**Methods:**

The evidence was taken from a systematic map of the impacts of chemicals arising from human activity on tropical reef-building corals published in 2021. All studies in the map database corresponding to the knowledge cluster “Evidence on the ecotoxicological effects of chemicals on corals” were selected. To identify subsequently published literature, the search was updated using a subset of the search string used for the systematic map. Titles, abstracts and full-texts were screened according to the criteria defining the selected cluster of the map. Because the eligibility criteria for the systematic review are narrower than the criteria used to define the cluster in the systematic map, additional screening was performed. Studies included were critically appraised and each study was rated as low, unclear, medium, or high risk of bias. Data were extracted from the studies and synthesised according to a strategy dependent on the type of exposure and outcome.

**Review findings:**

The systematic review reports the known effects of chemical exposures on corals from 847 studies corresponding to 181 articles. A total of 697 studies (161 articles) were included in the quantitative synthesis and 150 studies (50 articles) in the narrative synthesis of the findings. The quantitative synthesis records the effects of 2706 exposure concentrations-durations of 164 chemicals or mixtures of chemicals, and identifies 105 toxicity thresholds corresponding to 56 chemicals or mixtures of chemicals. When toxicity thresholds were compared to reference values set for the protection of marine organisms by environmental agencies, the reference values appear to be protective of corals for all but three chemicals assessed: the metal copper and the pesticides diuron and irgarol 1051.

**Conclusions:**

This open-access database of known ecotoxicological effects of chemical exposures on corals can assist managers in the ecological risk assessment of chemicals, by allowing easy determination of various ecotoxicological thresholds. Several limitations of the toxicity tests synthesised here were noted (in particular the lack of measurement of effective concentrations for more than half of the studies). Overall, most of the currently available data on coral toxicity should be replicated independently and extended to corals from less studied geographical regions and functional groups.

**Supplementary Information:**

The online version contains supplementary material available at 10.1186/s13750-023-00298-y.

## Background

Tropical coral reefs are among the most biologically rich ecosystems on Earth and are often compared to the rainforests of the oceans [1, 2]. They also provide substantial ecosystem services and goods with a net benefit of $30 billion per year [3] and contribute to the livelihoods of millions of people around the world [4, 5]. However, a wide range of anthropogenic stressors are leading to a steady decline in the world's coral reefs and jeopardises the benefits derived from their services and goods [4, 6, 7]. Reefs are subject to both global threats such as ocean warming [8] and local threats such as excessive sedimentation, overfishing, nutrient and chemical pollution from poor land management, agriculture and industry [9, 10].

**Fig. 1:**
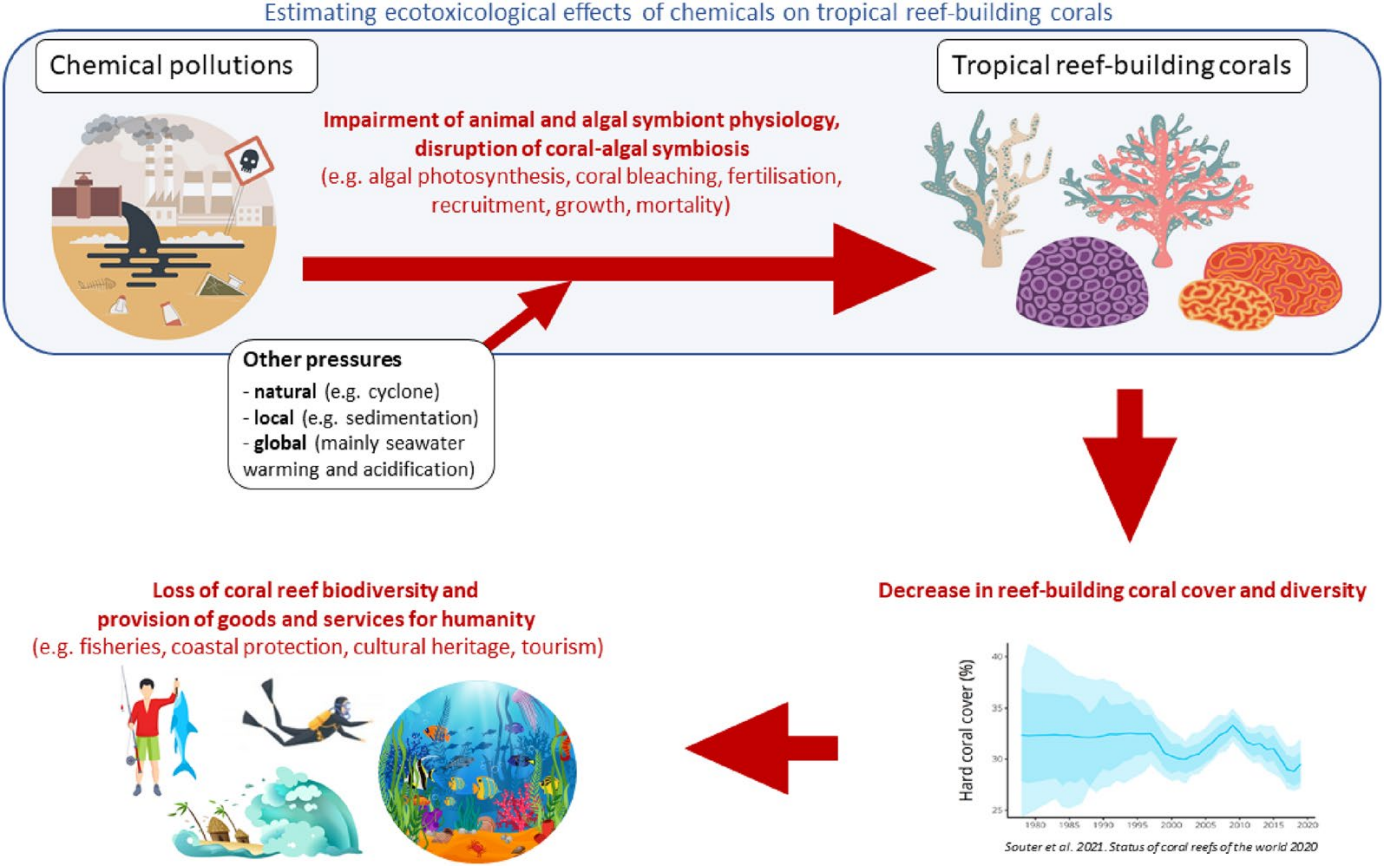
Conceptual model illustrating how the cascading ecotoxicological effects of seawater chemical pollution on tropical reef-building corals lead to the loss of coral reef biodiversity and less provision of goods and services to humanity. Images designed by Freepik

These threats are especially endangering scleractinian corals (hermatypic corals, sensu [11]), which are the main reef builders and form the three-dimensional structure of reefs that serve as habitat, food, and nurseries for thousands of other reef organisms [12]. Between 2009 and 2018, the average global population of scleractinian corals declined from 33.3% to 28.8%, which is equivalent to the loss of all scleractinian corals currently living in Australian coral reefs [13]. The vast majority of these corals live in association with endosymbiotic dinoflagellates (family Symbiodiniaceae, microalgae historically referred to as “zooxanthellae”) [14]. Symbiodiniaceae are critical to coral health because they photosynthesize and convert inorganic nutrients dissolved in seawater into organic molecules that are passed on to the host for its own energy needs. However, this symbiotic relationship is disrupted when corals are exposed to environmental stress. In particular, seawater warming is the main factor leading to coral bleaching, the breakdown of the coral-dinoflagellate symbiosis [15]. Since Symbiodiniaceae are the main food source of corals, bleaching can lead to coral death. Mass coral bleaching, which affects the vast majority of coral species within a reef, can in turn affect the functions of the entire ecosystem [12]. Local stressors, such as overfishing and land source water pollution add another stress to corals, as they reduce coral resistance and resilience to thermal stress [16–18]. Coastal water pollution is also a major threat per se [19], and has direct and indirect toxic effects on coral organisms and microalgae. Depending on the pollution type, the host, symbionts or both partners are impacted, through reduced calcification, photosynthesis or fecundity, as well as enhanced bleaching and oxidative stress, among other effects [20–22]. Water pollution also increases the incidence of coral diseases and pathogens [23], leading to severe decline in coral cover and reef functions ([23], Fig. 1). Declining water quality is therefore recognized as one of the greatest threats to coral health, but it is now recognized that management measures can aid in building ecosystem resilience to climate change [24].

The identification of the relative risk posed by pollutants to coral health, can be done through risk assessment. The goal of these risk analyses is to quantitatively and qualitatively determine the probability that a pollutant, at a defined concentration, will impact the physiological state of corals. This requires an assessment of the effects (or hazard assessment), for which the main inputs are the results of toxicity tests, expressed as measurement endpoints or *criteria for effects*, such as the No Observed Effect Concentration (NOEC) or Lowest Observed Effect Concentration (LOEC) for chronic exposure tests, or the median lethal (LC50) or median effective concentrations (EC50) for short-term exposure tests [25].

In this paper, we aim to systematically review experimental studies evaluating the toxicity of chemical pollutants to tropical reef-building corals to produce accessible and usable knowledge and data that can be used to calculate measurement endpoints in ecological risk assessment. From the quantitative estimates of effects, we determined toxicity thresholds as the highest exposures tested at which no statistically significant adverse effects were observed, and we compared them to regulatory predicted no effect concentrations for the protection of marine organisms, to assess whether these reference values are indeed protective of corals. To our knowledge, no such review exists yet, except the recent systematic review published in 2021 by Nalley et al. [26] but in which the pollutant categories oil, oil dispersants and microplastics were not considered. There are also existing reviews that focus on the toxicity of one or more categories of chemicals for corals, for example pesticides, industrial pollutants, hydrocarbons and metals [27], photosystem II herbicides [28], petroleum hydrocarbons [29], or organic ultraviolet (UV) filters [30]. Except for the 2021 reviews by Mitchelmore et al. [30] and Nalley et al. [26], none of these reviews mentions the method used to collect the studies, so they are not reproducible and the risk of bias due to the selection of certain studies cannot be assessed.

## Topic identification and stakeholder input

Coral reefs in French Overseas Territories cover 14,280 km^2^ representing 5% of the total coral reef area in the world [31, 32]. France is the country with the 4th largest coral reef area in the world, after Indonesia (18% of the world total area), Australia (17%) and the Philippines (9%) [32], and therefore has a major responsibility for coral reef conservation. In territories subject to intense demographic pressure and increasing anthropisation, the majority of coral reefs are degraded [33]. The French Ministry of Ecology has launched a project to assess the impact of chemical pollutants and nutrients on coral reefs and to improve coral reef protection and management at the national level. The project includes a systematic review to collect and analyse existing knowledge on the effects of chemical pollutants on corals. To know the status of the available literature on this topic, the first step was to create a systematic map of the effects of chemicals arising from human activities on tropical reef-building corals. The systematic map was published in the Environmental Evidence Journal in 2021 [34]. A large amount of scientific literature was found (908 articles, 7,937 studies – up to March 2020) and four relevant knowledge clusters were identified: (1) evidence on chemical bioaccumulation by corals (2050 studies); (2) evidence on the effects of nutrient enrichment on corals (2496 studies); (3) evidence on the effects of human activities on corals without reference to specific chemicals (1127 studies); and (4) experimental evidence on the ecotoxicological effects of chemicals on corals (other than nutrient enrichment, 2007 studies). The project steering committee, including the French Ministry of Ecology, decided that a systematic review of the ecotoxicological effects of chemical pollutants on corals, based on the fourth knowledge cluster of the systematic map, should be conducted to generate the necessary input data for another part of the project, the ecological risk assessment.

## Objective of the review

### Primary question

The primary question is: What are the toxicity thresholds of chemical pollutants for tropical reef-building corals?

### Components of the primary question

The above primary question has the following key elements:

Population: all tropical reef-building coral species (hermatypic scleractinian species, *Millepora* species, *Heliopora* species and *Tubipora* species). All developmental stages are considered (mobile planula, fixed polyp), as well as all coral compartments including dinoflagellate symbionts “*in hospite*” and the microbiome.

Exposure: all geogenic (e.g. trace metals) and synthetic chemicals (e.g. diuron) for which exposure concentrations are known. Inorganic and organic dissolved nutrients (e.g. nitrate) are excluded.

Comparator: population not exposed to chemicals; population before chemical exposure.

Outcome: all outcomes related to the health status of tropical reef-building corals, from molecular level (e.g. gene expression, enzyme activities) to colony (e.g. photosynthesis, bleaching) and population level (e.g. mortality rate).

Type of study: all experimental studies i.e. where exposure is controlled by researchers, in the laboratory or in the field.

## Methods

The systematic review followed the Collaboration for Environmental Evidence Guidelines and Standards for Evidence Synthesis in Environmental Management [35] and the protocol was published in Environmental Evidence [36]. There were several deviations from the protocol in the assessment of study validity. First, the experimental design criterion, which only led to a risk of bias if the experimental design was unknown, which in fact was never the case, was removed. However, the experimental design was retained as a descriptor of the study. Second, the criteria “Is the experiment replicated?”, “Is a solvent control present?”, and “Have effective exposure concentrations been measured?”, that led to a low or medium risk of bias, were revised as leading to a low or unclear risk of bias. This also resulted in a revision of the overall risk of bias of the studies, which was revised to be low, unclear, medium or high. Finally, a slight rewording of the “Exclusion” criterion was made. Another deviation from the protocol occurred in the quantitative synthesis: because the majority (95%) of the toxicity thresholds identified by chemical, outcome, species and life stage were each obtained from a single article, the risk of publication bias and the influence of individual studies on the results are obviously very high, therefore this was described in a narrative fashion rather than illustrated by a graphical assessment. The systematic review follows the ROSES reporting standards [37] (see Additional file 1).

### Searching for articles

The evidence was taken from the published systematic map of the impact of chemicals derived from human activities on tropical reef-building corals [34]. For the map, literature was searched in two bibliographic databases (Scopus and Web of Science Core Collection, March 19, 2020), three search engines (CORE, July 7, 2020, Google Scholar and GreenFile, July 8, 2020), two dissertations repositories (September 15, 2020), 11 specialist websites (from April 21 to May 29, 2020) and a call for literature (July 13, 2020). The search string used for the systematic map was built through a scoping exercise in Web of Science Core Collection database, using terms describing population and terms describing exposure (see details and assessment of comprehensiveness in Additional file 2 in [38]). To describe exposure, a detailed list of all the chemicals was not feasible to establish because of their large number, so the following approach was adopted to capture all the chemicals that could have an impact on corals. Terms were listed according to four levels of increasing specificity: (i) generic terms (e.g. contamination, pollution, chemicals); (ii) pressures (e.g. sewage, runoff) and usages (e.g. consumer product, biocide); (iii) classes of chemicals (e.g. nutrient, metal, pesticide, cosmetic, detergent, microplastic, petroleum); and (iv) within some classes of chemicals (e.g. metal), specific chemicals identified based on expert knowledge and whose impacts have been particularly studied in tropical corals (e.g. nickel, copper).

A search update was conducted to identify subsequently published literature. It followed the same protocol as described in the systematic map protocol [36, 38], except that (i) the search was limited to the period beginning in 2020; (ii) a subset of the search string was used; and (iii) no call for literature was made (see Additional file 2 in [36] for details and a comparison between the search strategy used for the systematic map and the search update used for the systematic review). The search string used for the systematic map was adjusted to fit the scope of the systematic review, which is narrower than the map. Specifically, the term “nutrient$” has been removed, as well as the generic terms “contamin*” and “pollut*”, and all terms describing pressures (e.g., sewage, runoff). The search string for the search update is as follows (Web Of Science format):

TS = (coral$ AND (toxicant$ OR chemical$ OR biocide$ OR "industrial product$" OR "consumer product$" OR "household product$" OR "biocidal product$" OR disinfect* OR oil OR metal$ OR pesticide$ OR herbicide$ OR insecticide$ OR fungicide$ OR antifoul* OR anti-foul* OR organochlorine$ OR "flame retardant$" OR detergent$ OR "perfluorinated compound$" OR pharmaceutical$ OR "personal care product$" OR cosmetic$ OR PAH$ OR petroleum OR hydrocarbon$ OR microplastic$ OR nanoparticle$ OR nano-particle$ OR "endocrine disrupt*" OR "organic compound$" OR dispersant$ OR metalloid$ OR solvent$ OR petrochemical$ OR additive$ OR preservative$ OR plasticizer$ OR hormone$ OR "transformation product$" OR "degradation product$" OR byproduct$ OR by-product$ OR sunscreen$ OR "UV filter$" OR "ultraviolet filter$" OR antibiotic$ OR phthalate$ OR PCB$ OR cyanide$ OR chlordecone OR nickel OR copper OR zinc OR cadmium OR mercury OR iron)).

The search update was performed on January 3, 2022 for bibliographic databases (Scopus, Web of Science Core Collection), search engines (CORE, GreenFile, Google Scholar), dissertation repositories (ProQuest Dissertations and Theses, Open Access Theses and Dissertations, the French thesis repository) and on January 4, 2022 for specialised websites (Additional file 2). Full details of the search update (sources, search strings used for the different sources, list of citation indexes of the Web of Science Core Collection) can be found in Additional file 2.

### Article screening and study eligibility criteria

#### Screening process

First, the 2,007 studies corresponding to the systematic map cluster four “*Evidence on the ecotoxicological effects of chemicals on corals*” [34] were selected (a study being the combination of a taxon, an exposure, and an outcome) and the cluster was updated by adding studies published since the map was produced. To this end, articles found during the search update were screened for cluster eligibility in two successive stages: first by title and abstract, and then by full-text. Articles for which the eligibility status was unclear during the screening of title and abstract were considered for the screening of full-text. Articles without abstracts that were selected based on their title were screened directly on full-text. Screening was performed by an experienced reviewer who had participated in all stages of screening and metacoding for the systematic map and whose decisions had therefore already been checked (2,148 of 15,177 titles and abstracts (14.2%) and 180 of 2,700 full-texts (6.7%) were independently screened by four reviewers and all disagreements discussed and resolved; and 20 out of 908 articles (2%) were independently coded by six reviewers and all discrepancies discussed and resolved; [38]). This screening can thus be considered as a continuation of the screening and metacoding for the systematic map. The reviewer never had to screen his/her own articles, except for the systematic map article [34] and its protocol [38] and the protocol for this systematic review [36], which were directly excluded during title and abstract screening. The list of articles from the search update that were rejected during full-text screening or whose eligibility status was unclear can be found in Additional file 3 with the reasons for exclusion or an explanation of why they could not be classified.

Second, because the eligibility criteria for the systematic review are narrower than those used to define the cluster in the systematic map (see “Eligibility criteria” section), additional screening was performed. Each excluded study was double-checked by a different reviewer from the review team. We ensured that the reviewers never had to screen or check their own articles. The list of rejected studies with the reasons for exclusion can be found in Additional file 3.

#### Eligibility criteria

Eligibility was assessed using the criteria listed in Table 1. The eligibility criteria for the systematic review, in addition to those used to define the cluster in the systematic map are (i) reference to exposure concentrations, (ii) existence of an unexposed population, and (iii) chemical exposure that can be dissociated from other physical disturbances (e.g. sedimentation/macroparticles).

**Table 1:** Eligibility criteria

Include	Exclude
*Population*	
- All tropical reef-building coral species (hermatypic scleractinian species, *Millepora* species, *Heliopora* species and *Tubipora* species) living in the shallow and the mesophotic zones. All developmental stages are considered (mobile planula, fixed polyp), as well as all coral compartments including dinoflagellates symbionts “in hospite” and microbiome	- Cold-water or deep-water corals - Ahermatypic corals - Free-living dinoflagellates (not hosted as symbionts within corals) - Studies conducted in coral reefs but not about corals (e.g. about coral reef fishes)
*Exposure*	
- All geogenic (e.g. trace metals) and synthetic chemicals (e.g. diuron) for which the exposure concentration is known - Exposure to a chemical alone or in combination with another chemical	- Studies assessing the impact of nutrients (e.g. nitrate) or eutrophication - Studies assessing the impact of human activities (e.g. river discharge, distance to a dump or to an industrial effluent source, tourism) on corals without reference to specific chemicals - Studies in which exposure to a chemical cannot be dissociated from other physical disturbances (e.g. sedimentation/macroparticles)
*Comparator*	
- Studies comparing population exposed to chemicals and control population unexposed to chemicals - Studies comparing population exposed to chemicals and population prior to exposure to chemicals (before/after) - For chemicals dissolved in a solvent, exposition to the solvent only was considered as control unexposed population	- Studies comparing population exposed to a range of concentrations/levels of chemicals in the absence of an unexposed population in the experiment
*Outcome*	
- All outcomes related to the health status of tropical reef-building corals, from the molecular (e.g. gene expression, enzyme activities) to the colony (e.g. photosynthesis, bleaching) and the population level (e.g. mortality rate) - Studies assessing impacts on coral symbionts/microbiome	- Studies reporting evidence of ingestion, concentration or accumulation/uptake of chemicals (bioaccumulation)
*Language*	
All articles written in English or French (in case a title or an abstract could not be found in English or French, it was directly screened on full-text)	
*Type of document*	
Journal article, book chapter, report, conference proceeding article, PhD or MSc thesis	Presentation, editorial material, letter or news item, conference or meeting abstract (i.e. very short summary), poster
*Type of content*	
In-situ or ex-situ experimental studies	Observational studies (field surveys), reviews and meta-analyses, modelling studies without experimental data

### Study validity assessment

The studies were critically appraised using the criteria described in Table 2. These criteria were based on the framework proposed by Vandenberg et al. [39] for the evaluation of the internal validity of experimental studies, and the knowledge of the experts in the review team (experts in ecotoxicology, coral ecotoxicology, coral biology and ecology, and chemical risk assessment) (see Additional file 4 for details). Two methodological issues raised by Mitchelmore et al. [30] were also considered (“exposure” source of bias in Table 2). The criteria “Performance”, “Detection” and “Exclusion (or attrition)” could only be assigned a low or high risk of bias”. The criteria “Selection” and “Other” could be assigned a low, medium, or high risk of bias. And the criteria “Experimental” and “Exposure” could be assigned a low or unclear risk of bias (Table 2). Indeed, replicating the experiment allows detection of possible mistakes in the implementation of the experiment (an error in implementation usually happens only once), but an unreplicated experiment is not necessarily error-prone. Similarly, the lack of solvent control or measurement of effective concentrations is not a problem if the solvent has no effect or if the nominal and effective concentrations are not different.

**Table 2:** Critical appraisal criteria

Source of bias	Criteria	Low risk of bias	Medium risk of bias	High risk of bias	Unclear risk of bias
Experimental	Is the experiment replicated (at least one replicate; a replicate is not exposed to water in contact with the other replicates)	– Yes			– No – Unknown (assumed to be No)
Exposure	If a solvent is used, is a solvent control present?	– Yes – N/A			– No – Unknown (assumed to be No)
	Have effective exposure concentrations been measured?	– Yes			– No – Unknown (assumed to be No)
Selection	Are there differences at baseline between groups C and E? (including difference in exposure environment, difference in biological model, difference in the set of individuals allocated to each group)	– No – N/A (for BA design)	– Yes but the effect is controlled and null – for in situ studies: yes but an attempt to minimize differences is made	– Yes (detail) – Unknown (assumed to be Yes)	
Performance	Are there differences in the way groups C and E (or B and A) are treated throughout the experiment?	– No		– Yes (detail) – Unknown (assumed to be Yes)	
Detection	Are there differences in the way the outcomes of groups C and E (or B and A) are assessed?	– No		– Yes (detail) – Unknown (assumed to be Yes)	
Exclusion (or attrition)	Are there differences in the way individuals or observations from groups C and E (or B and A) are removed from the study?	– No		– Yes (detail) – Unknown (assumed to be Yes)	
Other	Is there another source of bias? (e.g. reporting bias, insufficient description of the methods, an unforeseen event that occurred during the experiment)	– No	– Yes minor (detail)	– Yes major (detail)	

*C* Control, *E* Exposed, *B* Before, *A* After, *N/A* non-applicable

The overall risk of bias for a study was defined as low if all criteria leading to a low risk of bias were met; unclear if at least one criterion led to an unclear risk of bias, while all others led to a low risk of bias; medium if at least one criterion led to a medium risk of bias, while all others led to a low or unclear risk of bias; and high if at least one criterion led to a high risk of bias (Table 2). In the narrative synthesis, the results of studies with low overall risk of bias were first synthesised, and then the results of studies with unclear, medium and high risk of bias were considered. The quantitative synthesis consisted of determining toxicity thresholds (TTs): for each chemical, outcome and species, the highest concentration—and longest duration tested at which no statistically significant adverse effect is observed was determined (see “Data synthesis and presentation” section). Toxicity thresholds were determined first considering studies with low overall risk of bias, and then additionally considering studies with an unclear, medium and high risk of bias.

The critical appraisal was performed by two reviewers who independently assessed a sample of studies (3.4%) and discussed any discrepancy to ensure consistency. In addition, all doubtful cases were identified during the assessment and double-checked by experts from the review team, and 17.5% of studies were double-checked by another reviewer. We ensured that reviewers never had to critically appraise their own articles. The results of the critical appraisal are included in Additional file 4.

### Data coding and extraction strategy

The variables listed in Table 3 were extracted from the selected studies (a study being the combination of a taxon, an exposure, and an outcome). These metadata were added to the already extracted or coded data for the systematic map (i.e. type of study, ISO 3166 country or territory name, geographic coordinates or location, exposure and outcome categories). In this step, studies were divided into study cases, corresponding to an individual concentration-duration tested in an experiment, unless there were no data to extract. Only studies described by the authors as testing the effect of chemicals without testing other stressors were extracted, with the exception of studies that tested the combined effects of chemicals and elevated temperature or low pH (see “Potential effect modifiers/reasons for heterogeneity” section).

**Table 3:** Extracted variables

Variable	Description
Experimental design	Description of the experimental design: Control-Exposure (CE), Before-After exposure (BA), Before-After-Control-Exposure (BACE)
Taxon	Name of the taxon (coded from the systematic map)
Population	Description of the exposed population (e.g. coral nubbin with length, larva with age, egg-sperm bundle)
Life stage	Developmental stage of the exposed population (adult, juvenile, larva, gamete)
Control	Description of the control
Solvent	Description of the solvent and concentration used if any
Exposure	Exposure coded from the systematic map with a more complete description if necessary
Nominal concentration	Nominal concentration with unit
Effective concentration	Concentration(s) actually measured with unit and time of measurement (e.g. at the beginning and/or the end of the experiment)
Duration	Duration of exposure with unit. If several durations are available for one given exposure concentration in a test, the longest duration was extracted
Type of system	The type of experimental system (e.g. petri dish, beaker, tank, microcosm, mesocosm, in situ)
Temperature	Mean seawater temperature during exposure in °C
pH	Mean seawater pH during exposure
Measured outcome	Outcome coded from the systematic map with a more complete description if necessary. Detail was provided here in case the outcome was measured on a different developmental stage than the one exposed
Time after exposure	Time when the outcome was measured after exposure ceased
Quantitative result (Extraction only for the outcomes related to coral mortality, growth, settlement, symbiont density and photosynthesis)	Sample size, mean, type and measure of variation of the mean (e.g. standard deviation) for the control and the exposed group
Narrative result (Extraction only for the exposure categories Detergent, Dispersant, Microplastic, Nanoparticle, Pharmaceutical, UV filter, and Other, and only for the outcomes that did not undergo extraction of quantitative results)	Description of a statistically tested result

The data extraction strategy depended on the outcome and exposure categories considered (Table 4). First, quantitative data were extracted from text, tables and figures for all exposure categories and for outcomes related to coral mortality, growth, settlement, fertilisation, symbiont density (bleaching) and photosynthetic performance. Coral mortality was considered at all stages of development (e.g. gamete, larva, colony). To report on coral growth, both skeletal growth and calcification rates were considered here. Measurements of tissue growth were not taken into account because they were not comparable to skeletal growth. In two articles [40, 41], the rate of calcification was measured using both the buoyant weight technique and the alkalinity change technique. In these cases, the measurements made using the alkalinity change technique were chosen as being more accurate. Larval metamorphosis or success of settlement were considered as a measure of settlement. However, the number of coral recruits was not recorded, since this variable includes survival and growth of settled individuals. Fertilisation was only considered for species with external fertilisation. Indeed, for brooding species with internal fertilisation, the number of planulae released, often during an extended period of time, cannot be strictly compared to a fertilisation rate. The following variables were considered as a proxy for symbiont density in case it was not measured directly, in order of relevance: chlorophyll concentration in coral tissue, percentage of bleaching, colour scores, grey or blue colour measurements, and the fast component of the delayed fluorescence integrated over time which was found to be correlated with bleaching [42]. When several of these variables were available for the same study, the first one mentioned in this list was chosen. This hierarchy, which was not specified in the protocol, was established at the beginning of the data extraction process. Indeed, the chlorophyll concentration per symbiont is obtained through an actual measurement, and is relatively stable and species specific. It may increase with depth (to compensate for the decrease in light), but within an ecotoxicological experiment, it is usually rather stable. Therefore, the symbiont density can easily be back calculated from the chlorophyll measurement. The percentage of bleaching, and colour scores or measurements are based on indirect visual assessments of coral pigmentation, which do not allow calculating the symbiont density. The fast component of the delayed fluorescence integrated over time is not a variable usually used as a proxy for symbiont density but was chosen based on the study reported in [42]. The following variables were considered as measures of the photosynthetic performance of symbionts, in order of relevance: gross photosynthesis, net photosynthesis, effective quantum yield (the quantum efficiency of photosystem II photochemistry in the light, ΔF/Fm’), light-adapted maximum quantum yield (the maximum efficiency of photosystem II photochemistry in the light, Fv’/Fm’), dark-adapted maximum quantum yield (the maximum quantum efficiency of photosystem II photochemistry, Fv/Fm), slow component of the delayed fluorescence, and the maximum relative electron transport rate (rETR max). When several of these variables were available for the same study, the first one mentioned in this list was chosen. This hierarchy, which was not specified in the protocol, was established at the beginning of the data extraction process. Indeed, gross photosynthesis represents the maximal capacity of the symbionts to fix carbon and acquire energy. On the contrary, net photosynthesis is the result of what has been produced (in total, e. g. gross photosynthesis) minus what has been respired, so it is not completely a proxy of the maximal photosynthetic capacities. The other proxies derived from PAM fluorometry (quantum yields, etc.) are more related to the functioning of the photosystem II of the symbionts. They can decrease while the rates of gross photosynthesis remain constant or vice versa and they are generally used as early signs of impairment of photosynthetic capacities.

**Table 4:** Summary of the data extraction and synthesis strategy

	Hydrocarbon, metal, pesticide	Detergent, dispersant, microplastic, nanoparticle, pharmaceutical, UV filter, and other
Growth, fertilisation, mortality, settlement, symbiont density, photosynthesis	Quantitative synthesis: extraction of quantitative results	Quantitative synthesis: extraction of quantitative results
All other outcome categories	Not included in synthesis: no data extracted	Narrative synthesis of the findings: extraction of narrative results

The package metaDigitise [43] in the R environment [44] was used to extract data from figures. For each case, the sample size, the mean and a measure of the variation of the mean (e.g. standard deviation) were extracted for both the control and the exposed group.

Besides narrative results were extracted for (i) the exposure categories Detergent, Dispersant, Microplastic, Nanoparticle, Pharmaceutical, UV filter, and Other, where the total number of studies was relatively smaller (which limited the extraction of quantitative results) than for the categories Hydrocarbon, Metal, and Pesticide [34]; and (ii) the outcomes that were not included in the extraction of quantitative results (Table 4).

During data extraction, the missing or unclear information was coded as such. Data extraction was performed by two reviewers in a sequential process by exposure category. Data from one category were extracted by one or two reviewers, then a portion of the cases extracted by one reviewer was double-checked by the other reviewer to ensure consistency (on average 18% of the cases, see Additional file 5 for details on data extraction checking results). Any discrepancies were discussed and resolved and experts from the review team provided advices. This allowed consistency to be checked throughout the extraction process. The two reviewers also discussed difficult cases together during the extraction process, and consulted experts from the review team when they felt it was relevant. All extracted data are included in Additional file 5.

### Potential effect modifiers/reasons for heterogeneity

The following potential effect modifiers were considered:

- Chemical concentration and duration of exposure, since the highest concentration and/or the longest exposure will have the most detrimental effects on corals (e.g. [45, 46]);
- Taxon exposed, for example massive corals are known to be more resistant to stressors than branching corals [47];
- Developmental stage exposed, for example early life-stages can display higher sensitivity to chemical exposure that adults [48];
- Seawater temperature during exposure, for example seawater warming can increase coral sensitivity to chemicals [49];
- Seawater pH during exposure, for example seawater acidification can increase coral sensitivity to chemicals [50].

Taxonomic group and life stage are the main biotic factors influencing sensitivity to chemical exposure [51], while seawater temperature and pH are among the main abiotic factors modifying the toxicity of chemical pollutants [51]. Temperature and pH were particularly selected given the current warming and acidification of the oceans [8].

### Data synthesis and presentation

First, the studies included in the systematic review were described in a narrative synthesis of the characteristics of each primary study. Studies included in the quantitative synthesis were also described separately by population, exposure and outcome studied.

Subsequently, the results were synthesised according to a strategy dependent on the type of exposure and outcome (Table 4). The review focuses on the quantitative synthesis performed for the outcomes related to coral mortality, growth, settlement, fertilisation, symbiont density (bleaching) and photosynthetic performance. In addition, a narrative synthesis of the findings of individual primary studies was conducted but only for those categories with relatively few studies and those outcomes that were not included in the extraction of quantitative results due to limited resources and time (Table 4).

#### Quantitative synthesis

The outcomes included in the quantitative synthesis have lethal and sublethal toxicity endpoints distributed throughout the entire coral life cycle and concern both the coral animal and its symbionts. Sample size, mean, and level of variation around the mean (standard deviation, standard error or confidence intervals) for the control and the exposed group had to be reported in order for the study to be included in the quantitative synthesis. In cases where measures of variation were not reported, they were estimated by data imputation using the available means and standard deviations of all studies with complete information [52], by outcome category. When a measure of variation was reported but it was unclear whether it was a standard error or standard deviation, it was assumed to be a standard error, as inappropriately assuming a standard deviation would have given an overconfident effect size. When only boxplots were provided, means and standard errors were calculated using the package metaDigitise [43].

An estimate of the effect size was computed for each case using the standardised mean difference (Hedges’ d, [53]):

1 $$\begin{array}{c}{d}_{i}=\left(\left({\overline{X} }_{treatmen{t}_{i}}- {\overline{X} }_{contr\mathrm{o}{l}_{i}}\right)/{S}_{poole{d}_{i}}\right)\times {J}_{i}\end{array}$$

where ${\overline{X} }_{treatmen{t}_{i}}$ is the mean for the exposed group, ${\overline{X} }_{contr\mathrm{o}{l}_{i}}$ is the mean for the control group, ${S}_{poole{d}_{i}}$ is the pooled standard deviation for the two groups and ${J}_{i}$ is a correction term for small sample size. A positive (or negative) ${d}_{i}$ means that the measured outcome is higher (or lower) in the exposed group than in the control group, and a null ${d}_{i}$ means that there is no difference between the exposed and the control groups. The pooled standard deviation is calculated as:

2 $$\begin{array}{c}{S}_{poole{d}_{i}}=\sqrt{\frac{\left({n}_{treatmen{t}_{i}}-1\right)\times S{D}_{treatmen{t}_{i}}^{2}+\left({n}_{contro{l}_{i}}-1\right)\times S{D}_{contro{l}_{i}}^{2}}{{n}_{treatmen{t}_{i}}+{n}_{contro{l}_{i}}-2}} \end{array}$$

and the correction term ${J}_{i}$ as:

3 $$\begin{array}{c}{J}_{i}=1-\frac{3}{4\times \left({n}_{treatmen{t}_{i}}+{n}_{contro{l}_{i}}-2\right)-1}\end{array}$$

where ${n}_{treatmen{t}_{i}}$, ${n}_{contro{l}_{i}}$, $S{D}_{treatmen{t}_{i}}$, and $S{D}_{contro{l}_{i}}$ are the sample size and the standard deviation for the exposed and the control group, respectively. The variance of ${d}_{i}$ is calculated as [54]:

4 $$\begin{array}{c}var\left({d}_{i}\right)=\frac{{n}_{treatmen{t}_{i}}+{n}_{contro{l}_{i}}}{{n}_{treatmen{t}_{i}}\times {n}_{contro{l}_{i}}}+\frac{{d}_{i}^{2}}{2\left({n}_{treatmen{t}_{i}}+{n}_{contro{l}_{i}}\right)} \end{array}$$

To determine the ecotoxicological effects of chemical pollutants on corals, 95% confidence intervals were computed for each estimate of effect size ${d}_{i}$ as ${d}_{i}\pm 1.96*\sqrt{va{r}_{i}}$, to determine whether each ${d}_{i}$ was statistically significantly different from zero. All exposure concentrations and durations were standardised and the data were summarized, when possible, by determining a toxicity threshold (TT) corresponding to the highest concentration—longest duration tested at which no statistically significant adverse effect was observed, compared to the control. The TT was determined by chemical, outcome, species and life stage under normal temperature and pH conditions, first considering studies with low overall risk of bias, and then additionally considering studies with an unclear, medium and high risk of bias. The impact of increasing temperature and acidification on TTs was assessed by determining TTs under conditions of high temperature (≥ 30 °C) and low pH (< 8, ca. the ocean global average pH value [55]) and comparing them to those determined under conditions of temperature < 30 °C and pH ≥ 8.

To determine TTs, a dose–response relationship is needed, i.e. when the effect on organisms becomes apparent with gradually increasing exposure (by increasing concentration and/or time). Estimates of effect size, ${d}_{i}$, were therefore ordered by increasing exposure concentration-duration for TT identification. When several studies tested different exposure concentrations-durations for a given chemical, outcome, species, life stage and temperature and pH conditions, they were considered together to assess the dose–response relationship. When both significant and non-significant effects were observed for the same concentration-duration, likely due to differences in experimental conditions not considered here (e.g. host or symbiont genotype, coral life history, seawater physico-chemical conditions, etc.), the concentration-duration was considered to produce significant effects (conservative approach). Dose–response relationships based on at least five different exposure concentrations-durations were here considered valid to determine the TT. When the tested exposure concentrations-durations had all no significant effect or all a significant effect on the organisms, the TT could not be determined. In these cases, an indication that the TT is greater than or equal to the highest concentration-duration tested or less than the lowest concentration-duration tested, respectively, was given. When less than five concentration-duration were tested for a given chemical, outcome, species, life stage and temperature and pH conditions, the TT could not be determined and no indication was given. When exposure to a mixture of chemicals was tested, the TT was not determined, except for hydrocarbon products (e.g. crude oil, diesel) and oil dispersants. Finally, in cases when the dose–response relationship was not monotonic (effects alternately significant or non-significant as exposure gradually increased), the TT was not determined.

The choice of the synthesis method was guided by our objective to determine the thresholds above which chemical pollutants are toxic to corals, and from a management perspective, we wanted to compare these toxicity thresholds with the regulatory values used to protect marine organisms and verify whether corals are indeed protected by these regulatory values, which are calculated by extrapolating (by applying an assessment factor) the results of toxicity tests usually performed on non-coral species (e.g. a primary producer, a primary consumer, most frequently a daphnia, and a secondary consumer such as a fish). The available regulatory values are the predicted no effect concentrations (PNECs). Therefore, we chose to determine TTs as the highest exposures tested at which no statistically significant adverse effects were observed, allowing for a simple and direct comparison to these PNECs. However, it should be noted that other ecotoxicity endpoints, such as the effect concentration at which 10% effect is observed compared to the control (EC10) or at which 50% effect is observed compared to the control (EC50), could have been estimated by modelling the dose–response relationship, but such an approach requires that a sufficient number of concentration levels are available, as the precision of the estimate depends more on the number and spacing of concentrations rather than on the sample size per concentration level.

#### Narrative synthesis

Statistically significant results of the categories listed in Table 4 were summarized in narrative tables and a narrative synthesis was written, distinguishing results from studies with low, unclear, medium and high risk of bias. Results that were reported but not statistically tested, were not included in the narrative synthesis of the findings. All studies that were not included in either the quantitative or the narrative synthesis of the findings are provided in Additional file 3 with a rationale for why they could not be included.

## Review findings

### Review of the descriptive statistics

**Fig. 2:**
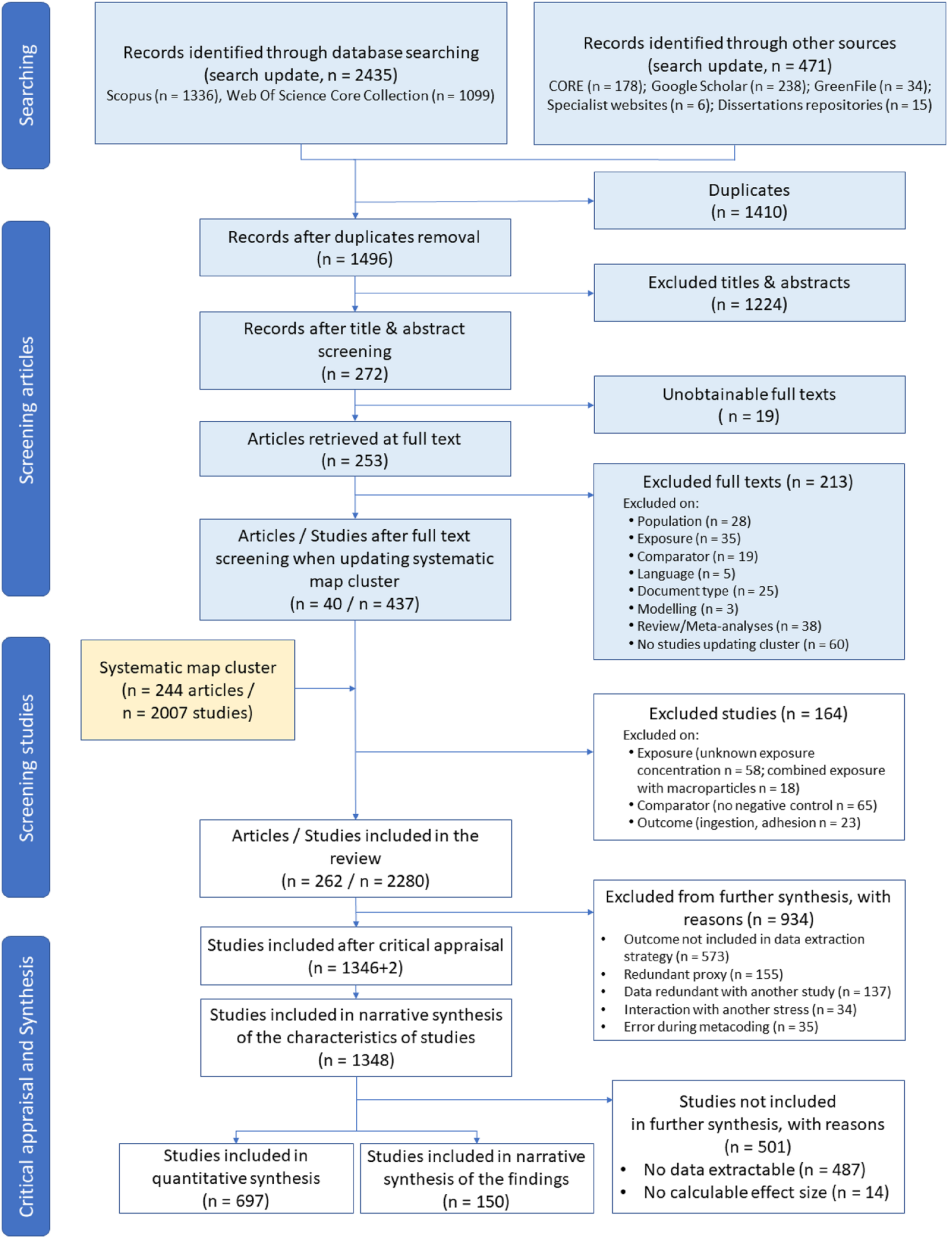
ROSES flow diagram [56] reporting the screening process of the articles from the search update (blue boxes) and of the studies from the cluster identified in the systematic map (yellow box). Two studies previously coded within two other studies in the systematic map cluster were separated at the critical appraisal stage

The search update returned 1336 records from Scopus and 1099 from Web of Science Core Collection. Additional sources gave 178 records from CORE, 238 from Google Scholar, 34 from GreenFile, 15 from dissertations repositories, and 6 from specialist websites (Additional file 2). The entire search resulted in a total of 2906 records reduced to 1496 after removing duplicates. Among them, 272 remained after title and abstract screening, and 253 of the 272 articles were screened on full-texts (19 full-texts could not be obtained). After full-text screening, 213 articles were excluded mostly because they were reviews/meta-analyses (17.8%, 38 articles), due to irrelevant exposure (16.4%, 35 articles) or population (13.1%, 28 articles), but also because studies did not meet the inclusion criteria for cluster four of the systematic map (28.2%, 60 articles, Fig. 2). A total of 40 articles were finally retained and added to the 244 articles of cluster four of the systematic map. As the eligibility criteria for the systematic review are narrower than those used to define the cluster in the systematic map, additional screening was carried out at the study level. A total of 164 studies were excluded, mainly due to the absence of negative control (39.6%, 65 studies) or due to unknown exposure concentration (35.4%, 58 studies, Fig. 2). This resulted in a total of 2280 studies (corresponding to 262 articles) answering the review question, but 934 studies were further excluded from synthesis, mostly because the outcome was not included in the data extraction strategy (see Table 4, 61.3%, 573 studies) or due to data redundancy (within study 16.6%, 155 studies, and between study 14.7%, 137 studies). In the end, 1348 studies were critically appraised and included in the narrative synthesis of the characteristics of studies (2 studies previously coded within 2 other studies in the systematic map cluster were separated at this stage). Of them, 697 studies (corresponding to 161 articles) were included in the quantitative synthesis and 150 (corresponding to 50 articles) in the narrative synthesis of the findings. The remaining 501 studies were excluded because no data were extractable or no effect size could be computed (Fig. 2). This systematic review, therefore, reports the findings of 847 studies corresponding to 181 articles. The lists of articles with unobtainable full-texts, excluded articles, and excluded studies are provided in Additional file 3 with reasons for exclusion.

### Description of the studies including study validity assessment

#### Source, language, document type

Most studies (83.8%) were found by searching publication databases (Scopus, Web of Science Core Collection). Other studies were retrieved through search engines (Google Scholar, CORE, GreenFile, 9.7%), dissertation repositories (OATD, 4.1%) and specialised websites (ReefBase, IFREMER, Ecotox knowledge base of the USEPA, 2.4%). Almost all studies are in English (99.3%), with a few in French (0.7%). Studies are mainly from journal articles (84.6%), then PhD theses (7.5%), reports (3%), conference proceedings (2.6%), master theses (2.2%) and book chapters (0.1%).

#### Geographical range

**Fig. 3:**
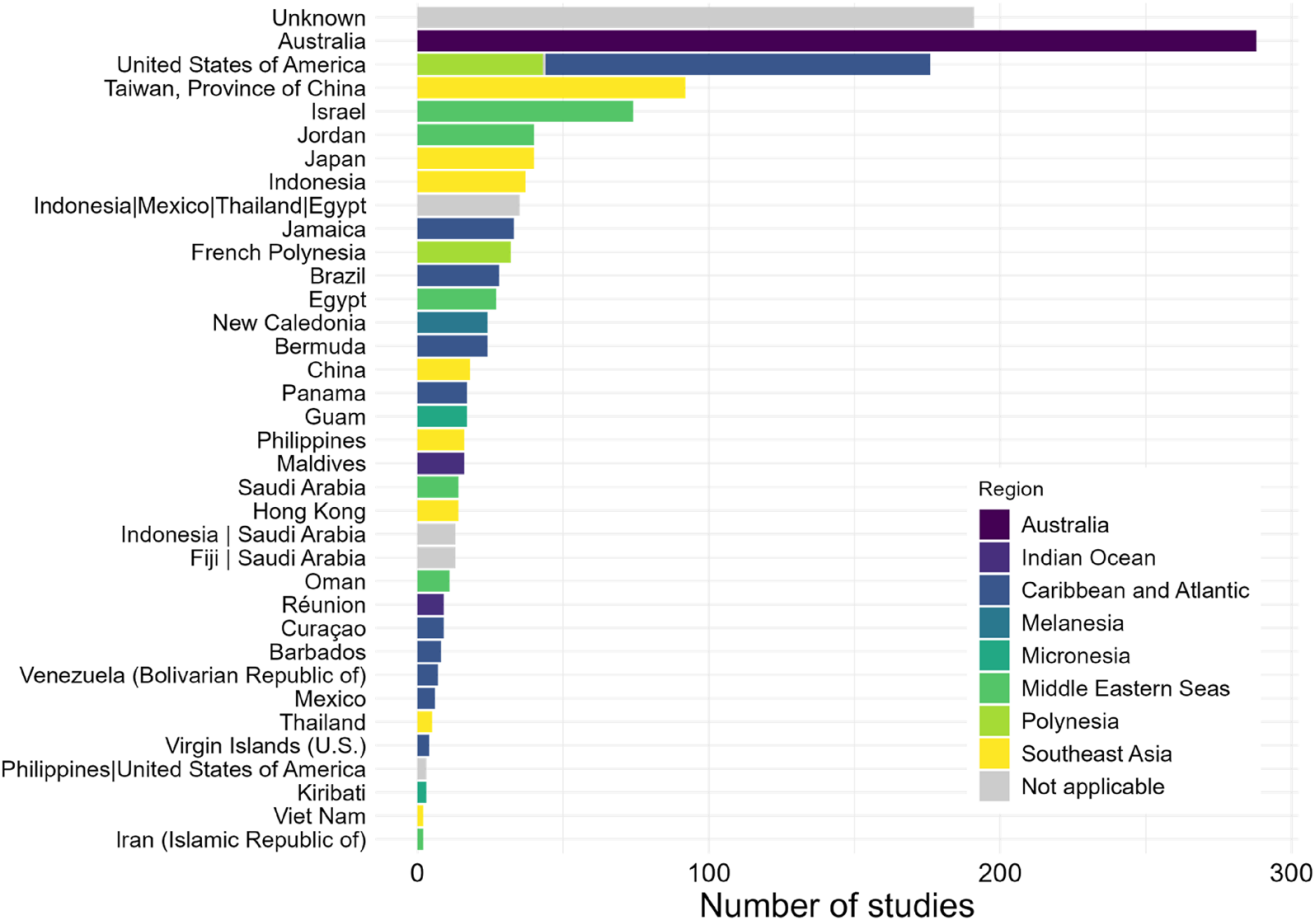
Distribution of studies (total 1348) by country of origin of corals (ISO 3166 country or territory names). Where corals came from more than one country, these countries are separated by a vertical bar. The different colours represent the country regions following [57] where the three Caribbean regions and the two Indian Ocean regions were grouped together

This systematic review focused on experimental studies, of which 91.6% were laboratory studies. This explains why the country of origin of the corals studied was unknown in a considerable number of studies (14.2%), since corals used in laboratory experiments were often from long-term propagated aquarium cultures. When the coral’s initial origins were provided, they were mainly from Australia (21.4%), the United States of America (13.1%), Taiwan (6.8%) and Israel (5.5%, Fig. 3).

#### Year of publication

**Fig. 4:**
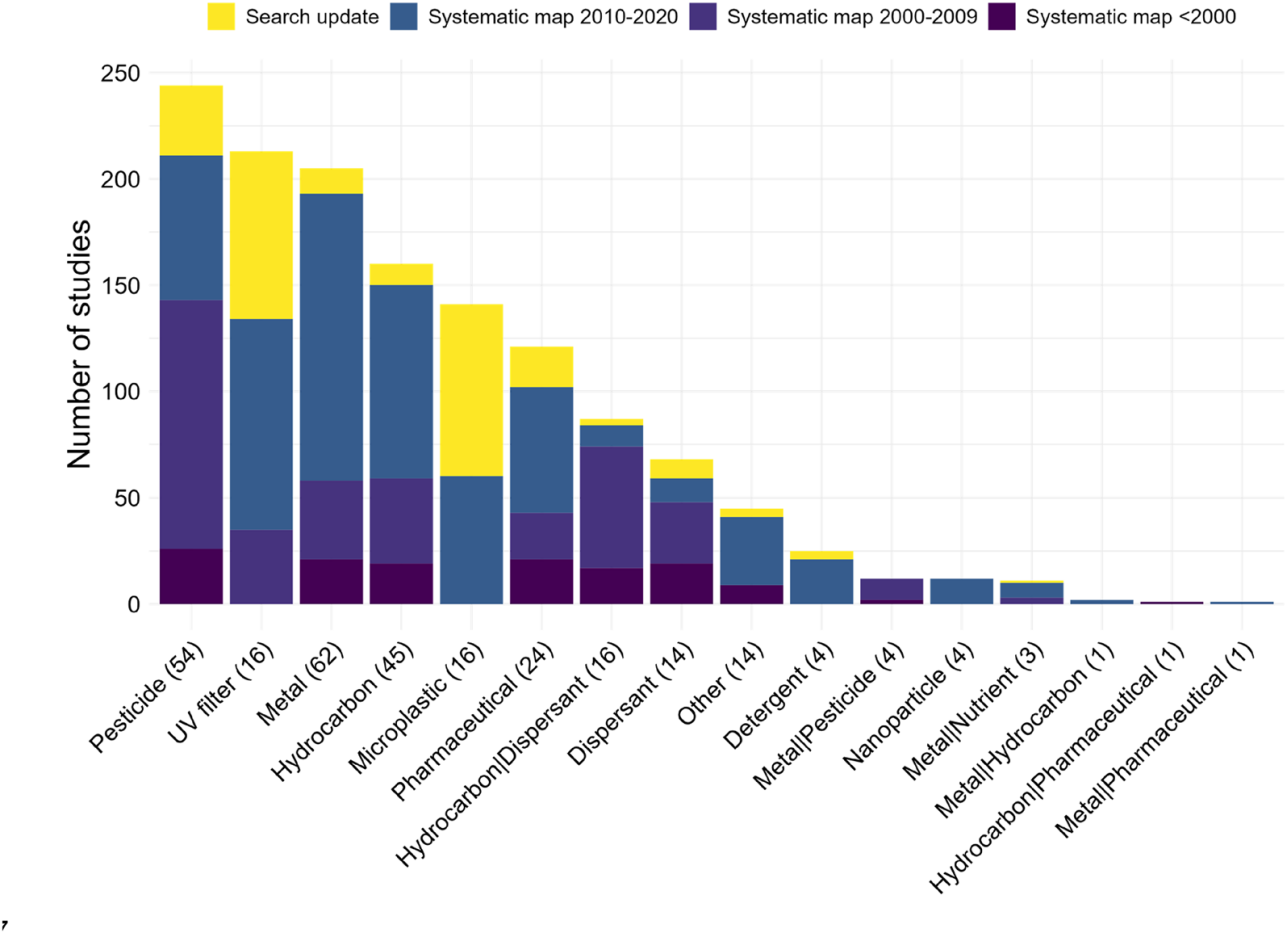
Distribution of studies (total 1348) by exposure category. The number of articles in each exposure category is indicated in brackets. The different colours indicate whether the studies are from the search update (period 2020–2022) or from the systematic map, in which case the publication period is also indicated

The 1348 studies selected for synthesis were mainly published after 2010 (62.7%, Fig. 4). Some exposure categories were investigated more recently, with a substantial number of studies coming from the search update addressing UV filters and microplastics (37.1% and 57.4%, respectively, Fig. 4).

#### Taxa studied

A total of 106 taxonomic units (+ “the reef-building corals” group) were identified in the 1348 studies selected for synthesis, with the ten most commonly studied species (or species complex) being mostly (9/10) fast-growing, branching species (*Stylophora pistillata*, *Pocillopora damicornis*, *Acropora millepora*, *Acropora tenuis*, *Acropora muricata*, *Seriatopora hystrix*, *Seriatopora caliendrum*, *Pocillopora verrucosa*, and *Acropora cervicornis*) and more rarely (1/10) the massive slow-growing *Porites astreoides* (Table 5). Focusing on the studies included in the quantitative synthesis, the ten most frequently studied species were the same as above but with a different ranking (Table 5).

**Table 5:** Total number of studies and number of studies in the quantitative synthesis and narrative synthesis of the findings for the 10 most studied taxa

Taxon	Total		Quantitative synthesis		Narrative synthesis	
*Stylophora pistillata*	180	(13.4%)	117	(16.8%)	20	(13.3%)
*Pocillopora damicornis*	155	(11.5%)	72	(10.3%)	28	(18.7%)
*Acropora millepora*	109	(8.1%)	79	(11.3%)	1	(0.7%)
*Acropora tenuis*	88	(6.5%)	65	(9.3%)	4	(2.7%)
*Acropora muricata*	66	(4.9%)	39	(5.6%)	8	(5.3%)
*Porites astreoides*	45	(3.3%)	33	(4.7%)	3	(2%)
*Seriatopora hystrix*	40	(3%)	23	(3.3%)	6	(4%)
*Seriatopora caliendrum*	39	(2.9%)	14	(2%)	2	(1.3%)
*Pocillopora verrucosa*	37	(2.7%)	21	(3%)	7	(4.7%)
*Acropora cervicornis*	34	(2.5%)	20	(2.9%)	6	(4%)

#### Exposure

Because data extraction and synthesis strategy varied by exposure category (Table 4), the total number of studies cannot be strictly compared across all categories. In terms of the number of studies in the quantitative synthesis covering six outcomes and all exposure categories, the metal category contains the most information (22.8% of the studies), followed by pesticides (20.7%), hydrocarbons (16.8%), UV filters (9.8%), microplastics (8%), and the combined exposure to hydrocarbons and dispersants (5.5%) (Table 6). The additional narrative synthesis of findings performed for exposure categories that contained relatively little information for quantitative synthesis included mainly studies on exposure to microplastics (33.3% of studies), UV filters (24%), and pharmaceuticals (20%).

**Table 6:** Total number of studies, and number of studies included in the quantitative synthesis and narrative synthesis of the findings, by exposure category

Exposure category	Total		Quantitative synthesis		Narrative synthesis	
Pesticide	244	(18.1%)	144	(20.7%)	Not included	
Metal	205	(15.2%)	159	(22.8%)	Not included	
Hydrocarbon	160	(11.9%)	117	(16.8%)	Not included	
UV filter	213	(15.8%)	68	(9.8%)	36	(24%)
Microplastic	141	(10.5%)	56	(8%)	50	(33.3%)
Pharmaceutical	121	(9%)	30	(4.3%)	30	(20%)
Dispersant	68	(5%)	31	(4.4%)	6	(4%)
Detergent	25	(1.9%)	11	(1.6%)	8	(5.3%)
Nanoparticle	12	(0.9%)	5	(0.7%)	4	(2.7%)
Other	45	(3.3%)	15	(2.2%)	16	(10.7%)
Hydrocarbon | dispersant	87	(6.5%)	38	(5.5%)	Not included	
Metal | pesticide	12	(0.9%)	10	(1.4%)	Not included	
Metal | nutrient	11	(0.8%)	11	(1.6%)	Not included	
Metal | hydrocarbon	2	(0.1%)	2	(0.3%)	Not included	
Metal | pharmaceutical	1	(0.1%)	0		Not included	
Hydrocarbon | pharmaceutical	1	(0.1%)	0		Not included	

Vertical bars (|) separate simultaneous exposure to several categories

Exposure to mixture of chemical categories (except the mixture of hydrocarbons and dispersants) and to nanoparticles and detergents were strongly under-studied (Table 6, Fig. 4).

#### Outcome

**Fig. 5:**
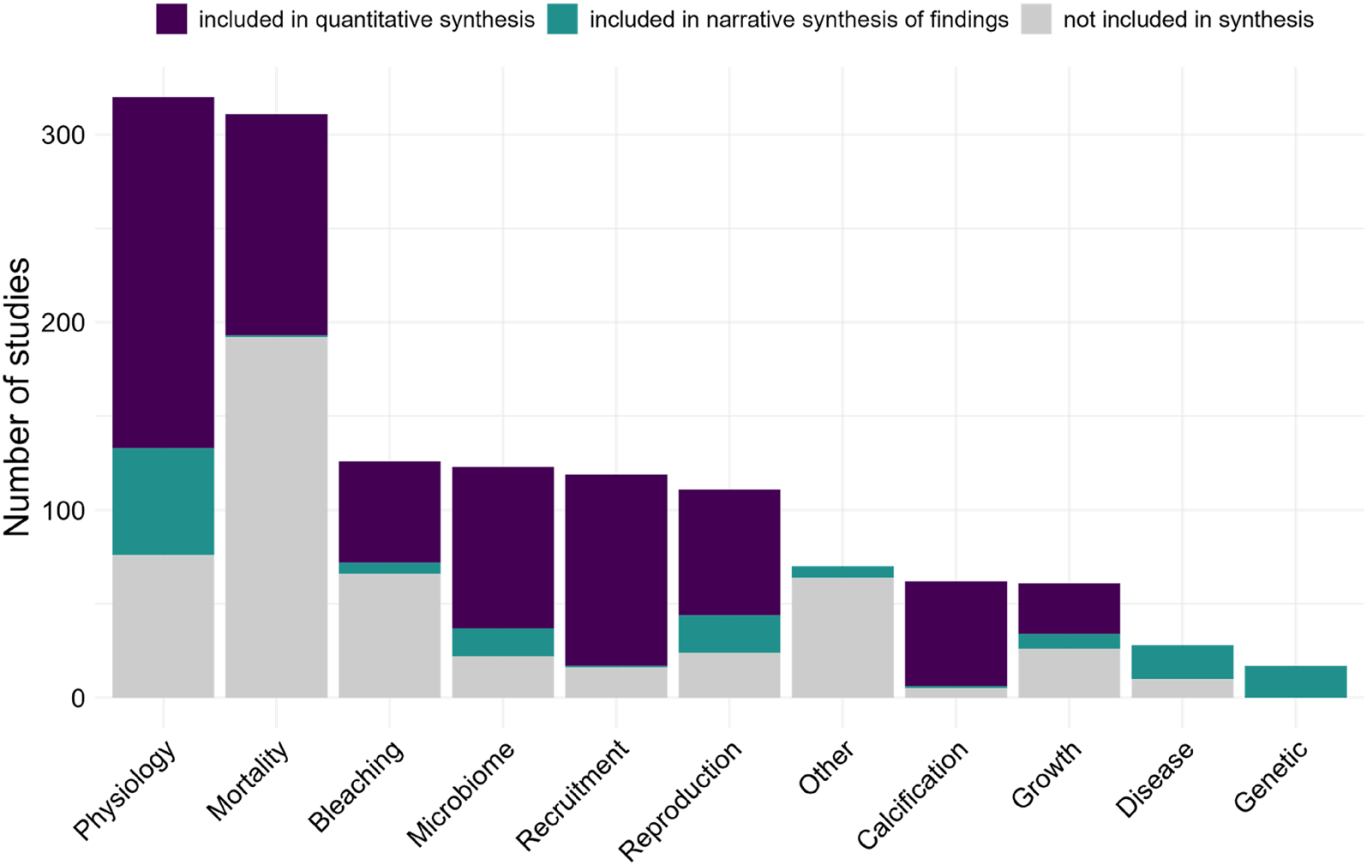
Distribution of studies (total 1348) by outcome category. The different colours indicate whether the studies are included in the quantitative synthesis (697 in total), in the narrative synthesis of findings (150), or not included in synthesis (501)

The most frequently measured outcomes were coral physiology (e.g. rate of photosynthesis, photosynthetic efficiency, respiration, enzyme activity, 23.7% of studies) and mortality (23.1%, Fig. 5). Of the 6 outcomes considered in the quantitative synthesis, symbiont photosynthetic performance was the most studied (Physiology category, 26.8% of studies), followed by symbiont density (Bleaching and Microbiome categories, 20.1%), mortality (Mortality category, 17.2%), settlement (Recruitment category, 14.6%), growth (Growth and Calcification category, 11.9%) and fertilisation (Reproduction category, 9.3%). In the narrative synthesis of findings, the most studied outcomes were those related to coral physiology (e.g. enzyme activity, respiration, 38% of studies) and to a lesser extent those related to reproduction (e.g. embryo to larva development, 13.3%), disease (e.g. signs of impaired health, 12%), genetics (e.g. gene expression, DNA lesions, 11.3%) and microbiome (e.g. microbiome community composition, 10%).

#### Study validity assessment

**Fig. 6:**
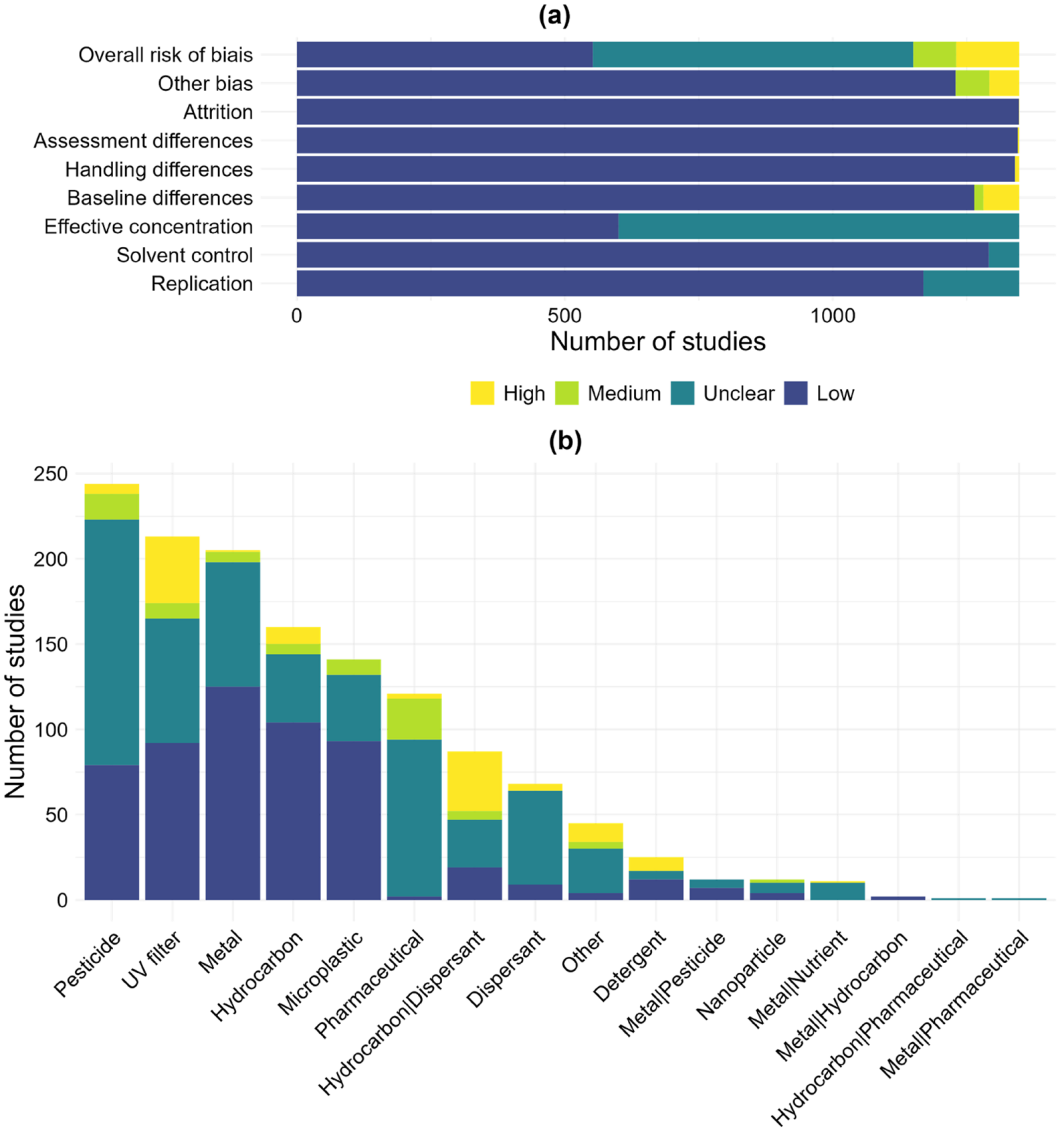
**a** Overall risk of bias of the 1348 studies and detailed risk of bias for each criterion (see Table 2 for a detailed description of criteria). **b** Overall risk of bias by exposure category

Of the 1348 studies selected for synthesis, 40.9% had an overall low risk of bias according to the criteria listed in Table 2, 44.4% had an overall unclear risk of bias, 5.9% had an overall medium risk of bias, and 8.8% had an overall high risk of bias (Fig. 6a). The unclear risk of bias was mainly due to the absence of information on effective exposure concentrations (unmeasured or unknown, 55.5% of studies) and/or lack of replication (13.3%) whereas the high risk of bias was mainly due to baseline differences between exposed and control groups (5% of studies) and/or other biases (e.g. inadequate description of methods, 4.2%, Fig. 6a). The risk of bias in the studies is not the same depending on the exposure category (Fig. 6b). The Metal category has a relatively high number of studies with low risk of bias while the Pharmaceutical category has almost none. The UV filter and Hydrocarbon & Dispersant categories have a relatively high proportion of studies with high risk of bias compared to other exposure categories.

### Data synthesis

#### Quantitative synthesis

##### Description of the study cases

**Fig. 7:**
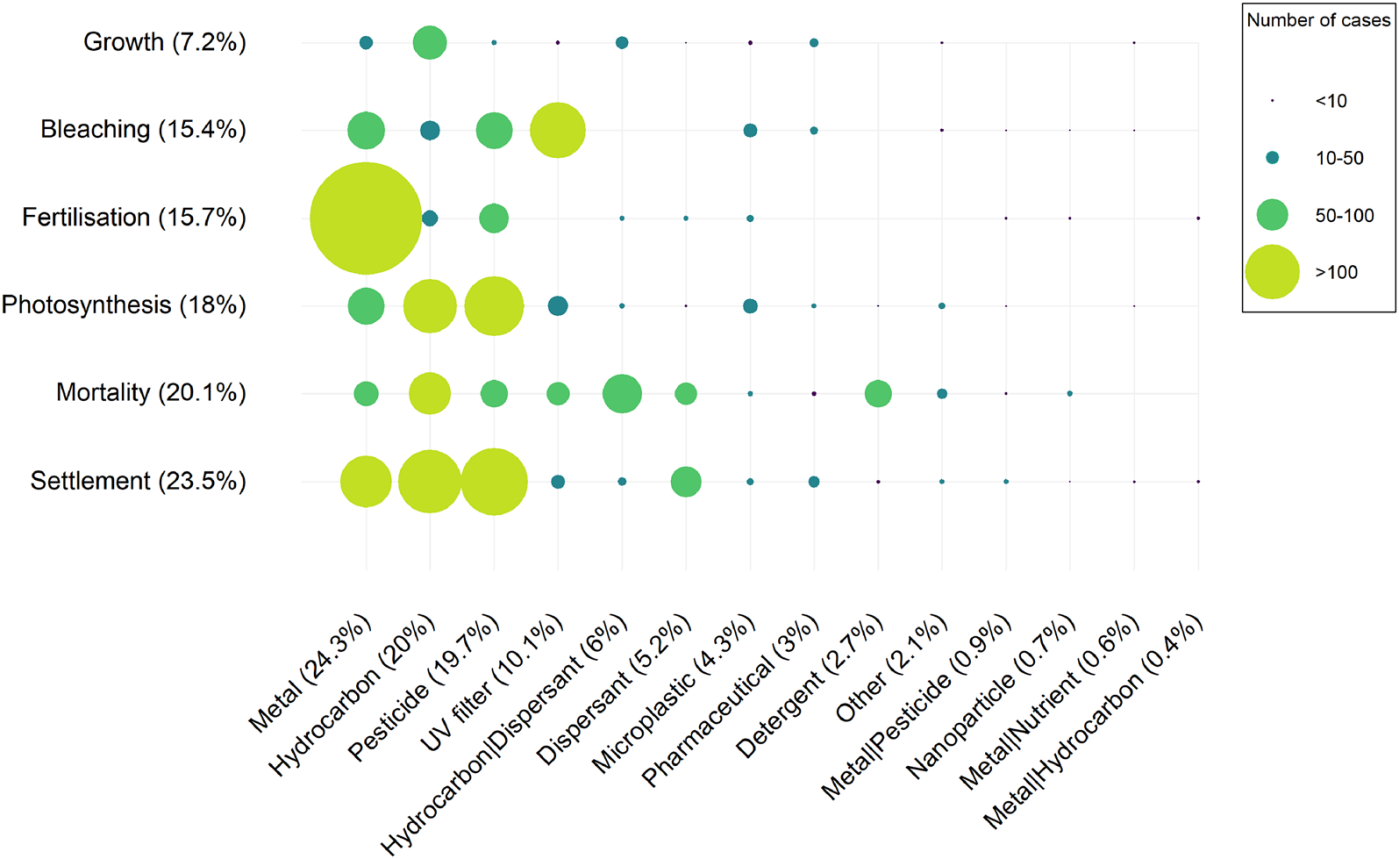
Heatmap showing the distribution and frequency of the 2706 study cases informing the quantitative synthesis into exposure categories and outcomes. The size of the circle is function of the number of study cases, and the proportion of cases in each exposure category and each outcome is indicated in brackets

The 697 studies selected for the quantitative synthesis were divided into 2897 cases, where a case is a single concentration-duration tested in a study. No effect size could be calculated for 126 of them, and 65 cases were related to the effect of the solvent, thus a total of 2706 cases were used for the quantitative synthesis. The most studied outcomes in terms of number of studies are not the same in terms of number of cases. Settlement has the highest number of cases (23.5%) followed by mortality (20.1%), symbiont photosynthetic performance (18%), fertilisation (15.7%), symbiont density (bleaching, 15.4%) and growth (7.2%, Fig. 7). Regarding exposure categories, the amount of information at the case study level is more or less the same as at the study level (Fig. 7, Table 6), with the Metal category having the highest number of study cases (24.3%). Some exposure categories preferably have more study cases for certain outcomes, such as fertilisation for metals, symbiont density (bleaching) for UV filters or mortality for detergents (Fig. 7).

A total of 164 different exposures were covered by the 2706 study cases in the quantitative synthesis (Additional file 5, “Cases quantitative synthesis” sheet, select study cases included in synthesis through the column “synthesis” and see the column “cat expo” describing exposure homogenised across studies). The exposures with the highest number of study cases (> 50) are exposure to copper (Metal category, 332 cases), diuron (Pesticide, 166), crude oil (Hydrocarbon, 109), 1-methylnaphtalene (Hydrocarbon, 80), polyethylene particles (Microplastic, 75), benzophenone-3 (UV filter, 66), fuel oil (Hydrocarbon, 65), phenanthrene (Hydrocarbon, 60), and lead (Metal, 55).

##### Determination of the toxicity thresholds

**Fig. 8:**
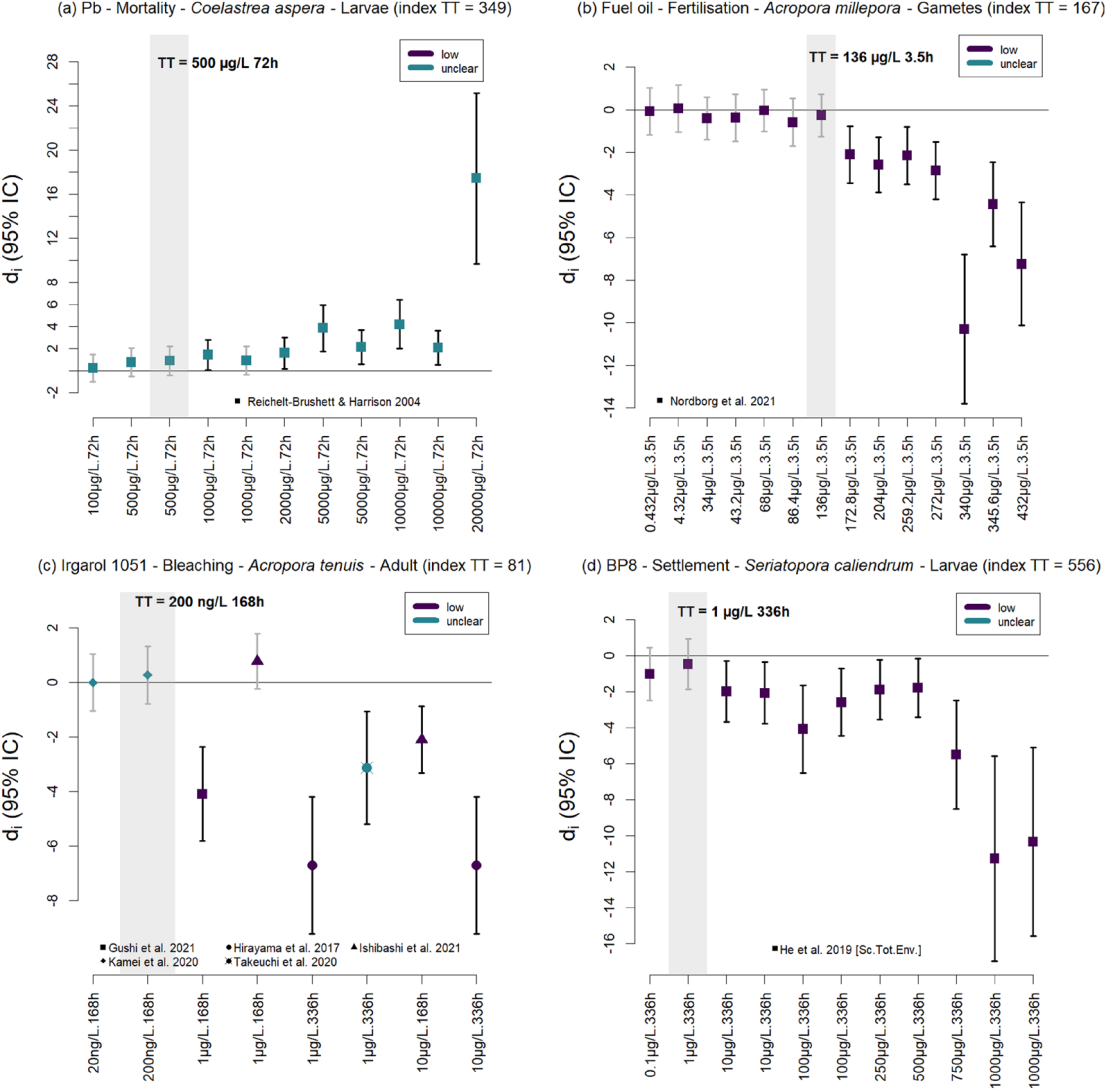
Examples showing how the toxicity thresholds were identified from effect size estimates (d_i_), by chemical, outcome, species, life stage, and temperature and pH conditions. Effect size estimates are ranked by increasing exposure intensity, with point symbols representing different primary research articles. The 95% confidence intervals of estimates indicating an adverse effect significantly different from zero are coloured black, while those that are not significant are grey. The dark and light blue points indicate studies with an overall low and unclear risk of bias, respectively. Index TT is the number given to each set of effect size estimates used to determine the toxicity threshold in Additional file 5 (“Cases quantitative synthesis” and “Toxicity thresholds” sheets)

The 2706 study cases corresponded to a total of 641 combinations of exposure, outcome, species, life stage, temperature and pH conditions, for which a toxicity threshold (TT) could be determined (Fig. 8, Additional file 5, see the index number given to each set of study cases (effect size estimates) used to determine toxicity thresholds in “Cases quantitative synthesis” and “Toxicity thresholds” sheets). Considering the differences in the overall risk of bias of the studies, these 641 combinations lead to 663 possibilities to determine a TT, but only 107 TTs, corresponding to 56 different exposures, could finally be determined (Table 7, Additional file 5). For those TTs that could not be determined, it was mainly because fewer than five concentrations-durations were tested (368), or because the exposure was a combination of chemicals (99) or the relationship was not monotonic (31). Regarding the risk of bias of the studies included in TTs calculations, 60 TTs were based on studies with a low risk of bias, 41 TTs were based on studies with an unclear risk of bias, 1 TT was based on a study with a medium risk of bias, and 5 TTs were based on a mix of studies having low and unclear or high risk of bias (e.g. Figure 8c).

**Table 7:** Toxicity thresholds (TTs) overview by exposure

	Metal	Hydrocarbon	Pesticide	UV filter	Dispersant	Microplastic	Pharmaceutical	Detergent	Other	Nanoparticle	Hydrocarbon | dispersant	Metal | pesticide	Metal | nutrient	Metal | hydrocarbon	Total
Study cases	658	542	532	274	140	116	81	73	58	19	162	24	15	12	2706
Combi-nations	139	118	136	58	31	50	30	11	23	7	37	10	11	2	663
TTs [low]	30 [21]	29 [24]	24 [6]	7 [5]	9	0	0	4 [4]	2	0	0	0	0	0	105 [60]
Exposure	Al (2), Cd (1), Cu (15), Fe (2), Ga (2), Mn (1), Ni (1), Pb (4), V (2)	1-methylnaphthalene (7), Anthracene (1), Benzene (1), Crude oil (5), Fuel oil (5), gas Condensate (1), mineral derived lubricant oil (1), Naphthalene (1), Phenanthrene (2), production formation water (1), p-Xylene (1), Toluene (1), Vegetal derived lubricant oil (1), Weathered condensate (1)	Carbaryl (1), Chlorothalonil (1), Chlorpyrifos (1), Chlorpyrifos oxon (1), Cyanide (2), Diazinon (1), Diuron (4), Endosulfan (1), Fipronil (1), Imidacloprid (1), Irgarol 1051 (3), MEMC (2), Permethrin (1), Profenofos (1), Propiconazole (1), Tributyltin (2)	Benzophenone-1 (1), Benzophenone-2 (1), Benzophenone-3 (2), Benzophenone-8 (3)	Ardrox 6120 (1), Corexit 9500 (1), Corexit 9527 (1), Dispolen 36 S (1), Emulgal C 100 (1), Finasol OSR 52 (1), Inipol 90 (1), Slickgone LTSW (1), SlickgoneNS (1)			Linear alkylbenzene sulfonate (2), Nonylphenol ethoxylate (2)	DMSO (1), Ethylene glycol (1)						56

For each exposure category (vertical bars (|) separate simultaneous exposure to several categories): (i) number of study cases; (ii) number of combinations between study overall risk of bias, exposure, outcome, species, life stage, temperature and pH conditions, for which a toxicity threshold might be determined; (iii) number of TTs identified (the number of TTs determined from studies with an overall low risk of bias is indicated within square brackets); and (iv) list of the exposures for which a TT was determined (the number of TTs determined is indicated in brackets). The exposures for which at least one TT was determined based on studies with an overall low risk of bias are underlined

##### Influence of the overall risk of bias of studies on the toxicity thresholds

Of the 107 TTs identified, four TTs allowed an assessment of the effect of overall risk of bias of the studies on TTs. This was achieved by comparing the two TTs identified using only studies with a low risk of bias with the two TTs identified using all studies, regardless of their risk of bias. The comparison revealed that the TTs were the same regardless of the set of studies considered. This resulted in a final set of 105 TTs after these two duplicates were removed.

It should be noted that three TTs could be determined only based on low-risk-of-bias studies but not when all studies were considered (because the relationship was no longer monotonic), and that three TTs could be determined when all studies were considered but not when only low-risk-of-bias studies were considered (because there were fewer than five concentrations-durations available).

**Fig. 9:**
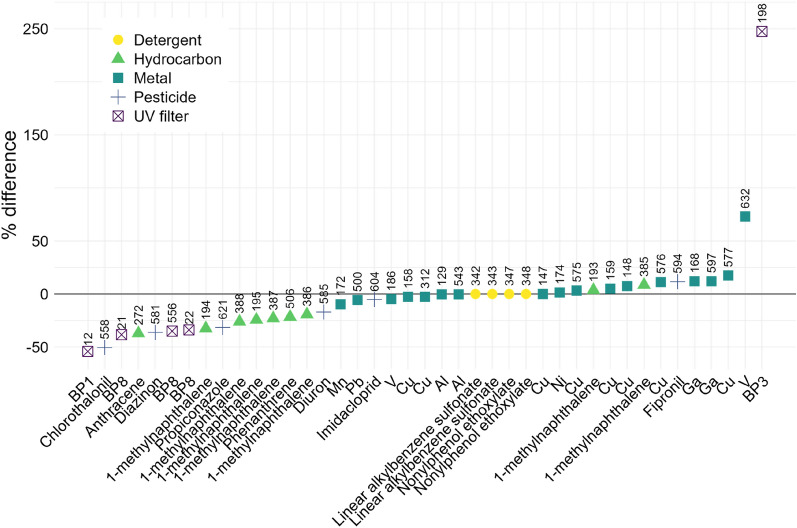
Differences between the toxicity thresholds of chemical pollutants calculated using their effective or nominal exposure concentrations (expressed as % of the nominal concentration; [effective – nominal]/nominal). Above each point is indicated the index number given to each set of study cases (effect size estimates) used to determine the toxicity threshold (Additional file 5, “Cases quantitative synthesis” and “Toxicity thresholds” sheets)

The influence of the studies’ overall risk of bias on the TTs is therefore difficult to assess. However, the value of the TTs can change substantially depending on whether the nominal or the effective exposure concentration is considered. Indeed, the example of the UV filters is striking as the effective concentrations can be up to 54% lower (e.g. benzophenone-1) or 247% higher (e.g. benzophenone-3) than the nominal concentrations (Fig. 9).

##### Publication bias and influence of individual studies on the toxicity thresholds

Because the majority (95%) of TTs identified by chemical, outcome, species and life stage were each obtained from a single article, the risk of publication bias, and the influence of individual studies on the results, are obviously very high. In particular, publication bias—the risk that unpublished, statistically non-significant results are not included—may have prevented us from determining TTs when all concentrations-durations had a significant effect, or when fewer than five concentrations-durations were available. Where TTs were determined, the impact of publication bias is limited. Indeed, if concentrations—durations tested in these supposedly non-included studies were below the TT, this has no impact because the TT is the highest concentration and longest duration tested at which no statistically significant adverse effect was observed. However, if exposure concentrations—durations above the TT were tested in these presumptively non-included studies, this implies that the TT may be underestimated, which has limited consequences from an environmental perspective (i.e., the TT is overly conservative).

##### Description of the toxicity thresholds identified

The 105 identified TTs correspond to 56 exposures. Copper is the chemical for which the most TT information was obtained, with 15 TTs covering four outcomes and eleven species. About two thirds of the exposures (37) had only one identified TT (Table 7). The 105 TTs correspond to 28 species, including *A. millepora* (28 TTs), *A. tenuis* (24 TTs) and *Poc. damicornis* (8 TTs), and more than half of the TTs involve early life stages (26 TTs for gametes and 44 TTs for larvae). The majority of the TTs (90) relates to normal temperature and pH conditions. Only two TTs can be compared between normal and high temperature conditions, with a lower TT for the effect of exposure to copper on *A. millepora* larval settlement under high temperature conditions (4 µg/L for 6 h at 32 °C) than under normal conditions (16 µg/L for 6 h at 28 °C). This suggests that corals may be more sensitive to chemical exposure when combined with thermal stress.

The exposure durations associated with TTs exposure concentrations are generally short: 71 TTs are calculated based on exposure durations of less than or equal to 24 h, and only 6 on exposure durations of more than 96 h. The existing studies thereby address short-term, acute ecotoxicological effects of chemical pollutants. The TTs exposure concentrations are mainly expressed in weight/volume, but 4 TTs are expressed in volume/volume (gas condensate, dispersant Dispolen 36S, Emulgal C100 and Inipol 90).

**Fig. 10:**
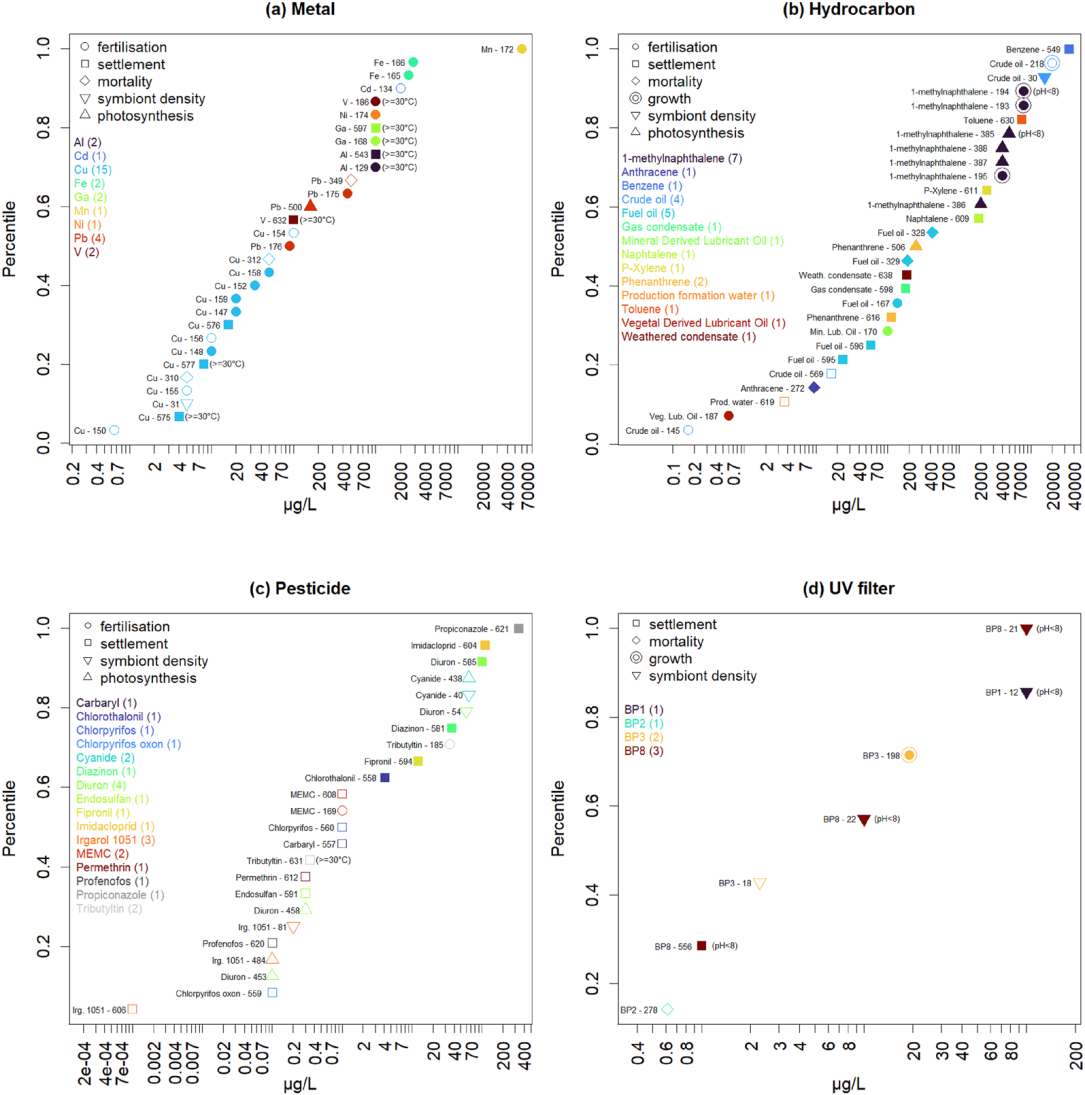
Cumulative distribution of toxicity threshold (TT) concentrations (µg/L) for all exposure belonging to the categories: **a** Metal (30 TTs), **b** Hydrocarbon (28 TTs), **c** Pesticide (24 TTs), and **d** UV filter (7 TTs). Point colours and types indicate the corresponding exposure (with total number of TTs indicated in brackets) and outcome, respectively. TTs based on studies with an overall low risk of bias are indicated by filled points, and those corresponding to high temperature or low pH conditions have this information indicated in brackets. On the left of each point is indicated the exposure with the index number given to each set of study cases (effect size estimates) used to determine the toxicity threshold (Additional file 5, “Cases quantitative synthesis” and “Toxicity thresholds” sheets). Nominal exposure concentrations are given here, unless only the effective concentration was available

For the metal category, the TT concentrations range from 4 µg/L (copper, settlement) to 60 mg/L (manganese, fertilisation) when only studies with an overall low risk of bias are considered, and from 0.65 µg/L (copper, fertilisation) to 60 mg/L (manganese, fertilisation) when all studies are considered (Fig. 10a). For the hydrocarbon category, TT concentrations range from 0.6 µg/L (vegetal derived lubricant oil, fertilisation) to 34.2 mg/L (benzene, settlement) when only studies with an overall low risk of bias are considered, and from 0.165 µg/L (crude oil, fertilisation) to 34.2 mg/L (benzene, settlement) when all studies are considered (Fig. 10b). For the pesticide category, TT concentrations range from 4.1 µg/L (chlorothalonil, settlement) to 333 µg/L (propiconazole, settlement) when only studies with an overall low risk of bias are considered, and from 1 ng/L (irgarol 1051, settlement) to 333 µg/L (propiconazole, settlement) when all studies are considered (Fig. 10c). For the UV filter category, TT concentrations range from 1 µg/L (benzophenone-8, settlement) to 100 µg/L (benzophenone-8, bleaching) when only studies with an overall low risk of bias are considered, and from 0.615 µg/L (benzophenone-2, mortality) to 100 µg/L (benzophenone-8, bleaching) when all studies are considered (Fig. 10d). For the dispersant category, no TTs could be based on studies with an overall low risk of bias and the TT concentrations range from 1 mg/L (Corexit 9527, fertilisation) to 5 mg/L (Slickgone NS, settlement). For the detergent category, all four TT concentrations are based on studies measuring mortality with an overall low risk of bias; they are equal to 0.75 mg/L for linear alkylbenzene sulfonate (*Poc. damicornis* and *Sty. pistillata*) and 1 mg/L for nonylphenol ethoxylate (*Poc. damicornis* and *Sty. pistillata*). Finally, for the “Other” category (i.e. the chemicals that could not be classified elsewhere), two TT concentrations were determined based on studies with an overall unclear risk of bias and which measured the mortality of coral tissue balls after exposure to DMSO (dimethyl sulfoxide) and ethylene glycol, both chemicals being used as cryoprotectants in studies not directly relevant to environmental ecotoxicology.

##### Comparison of the toxicity thresholds with reference values

The TTs obtained were contextualised by comparing, for each chemical, the lowest TT with the predicted no effect concentrations (PNECs) for the protection of marine organisms (environmental quality standard (EQS) protecting marine organisms, from the French National Institute for Industrial Environment and Risks (Ineris), https://substances.ineris.fr/fr/page/9). When these regulatory PNECs were not available, the PNECs for marine waters from the European CHemicals Agency (ECHA, https://echa.europa.eu) or from the NORMAN ecotoxicology database (https://www.norman-network.com/nds/ecotox/lowestPnecsIndex.php) were used instead. This comparison shows that the reference values appear to be protective for corals for all but three chemicals assessed: the metal copper and the pesticides diuron and irgarol 1051 (Table 8). It should be noted that this conclusion is supported by TTs based on studies having an unclear risk of bias, and considering nominal exposure concentrations.

**Table 8:** Toxicity thresholds (TTs) contextualisation

Chemical (CAS number) [index TT] [ref.]	Risk of bias	Outcome	T°C and pH	TT nom. [c] (µg/L)	TT eff. [c] (µg/L)	TT duration (h)	Ref. value (µg/L)	Source reference value	
Metal									
Al (7446–70-0) [TT 129] [58]	L	Fert	High T°	1000	996	3.5	No valid PNEC derived	ECHA (PNEC marine water)	
Cd (7440–43-9) [TT 134] [59]	U	Fert	Normal	2000	-	5.5	0.2	INERIS (AA-QS marine eco)	
Cu (7440–50-8) [TT 150] [60]	U	Fert	Normal	**0.65**	-	4	0.8	INERIS (AA-QS marine eco)	*
Fe (10421–48-4) [TT 165] [61]	L	Fert	Normal	-	2500	5.5	2.4	ECHA (PNEC freshwater /10)	
Ga (13450–90-3) [TT 168] [58]	L	Fert	High T°	1000	1120	3.5	Unknown		
Mn (7773–01-5) [TT 172] [62]	L	Fert	Normal	60,000	54,200	5.5	0.4	ECHA (PNEC marine water)	
Ni (7440–02-0) [TT 174] [63]	L	Fert	Normal	1000	1014	5.5	8.6	INERIS (AA-QS marine eco)	
Pb (7439–92-1) [TT 176] [59]	L	Fert	Normal	-	90	5.5	1.3	INERIS (AA-QS marine eco)	
V (7718–98-1) [TT 632] [58]	L	Settl	High T°	100	173	3.5	2.5	ECHA (PNEC marine water value for Vanadium CAS 7440–62-2)	
Hydrocarbon									
1-methylnaphthalene (90–12-0) [TT 386] [64]	L	Photo	Normal	2000	1614	48	0.12	NORMAN (PNEC marine water)	
Anthracene (120–12-7) [TT 272] [65]	L	Mort	Normal	9.4	5.914	48	0.1	INERIS (AA-QS marine eco)	
Benzene (71–43-2) [TT 549] [45]	L	Settl	Normal	–	34,237.94	24	8	INERIS (AA-QS marine eco)	
Naphtalene (91–20-3) [TT 609] [45]	L	Settl	Normal	–	1875	24	2	INERIS (AA-QS marine eco)	
P-xylene (106–42-3) [TT 611] [45]	L	Settl	Normal	–	2406.25	24	4.4	ECHA (PNEC freshwater /10)	
Phenanthrene (85–01-8) [TT 616] [65]	L	Settl	Normal	112.5	–	48	1.3	NORMAN (PNEC marine water)	
Toluene (108–88-3) [TT 630] [45]	L	Settl	Normal	–	7500	24	7.4–680	ECHA (PNEC marine water)	
Pesticide									
Carbaryl (63–25-2) [TT 557] [66]	U	Settl	Normal	1	-	18	0.023	NORMAN (PNEC freshwater /10)	
Chlorothalonil (1897–45-6) [TT 558] [67]	L	Settl	Normal	4.1	2.03	96	0.004	ECHA (PNEC marine water)	
Chlorpyrifos (2921–88-2) [TT 560] [66]	U	Settl	Normal	1	–	18	0.033	INERIS (AA-QS marine eco)	
Chlorpyrifos oxon (5598–15-2) [TT 559] [66]	U	Settl	Normal	0.1	–	18	0.0048	NORMAN (PNEC freshwater /10)	
Cyanide (151–50-8, 143–33-9) [TT 40] [68]	U	Bleach	Normal	65	–	3	0.2	ECHA (PNEC marine water)	
Diazinon (333–41-5) [TT 581] [67]	L	Settl	Normal	37	23.6	96	0.001	NORMAN (PNEC freshwater /10)	
Diuron (330–54-1) [TT 453] [69]	U	Photo	Normal	**0.1**	-	96	0.2	INERIS (AA-QS marine eco)	*
Endosulfan (115–29-7) [TT 591] [66]	U	Settl	Normal	0.3	-	18	0.0005	INERIS (AA-QS marine eco)	
Fipronil (120068–37-3) [TT 594] [67]	L	Settl	Normal	12.3	13.72	96	0.00007	NORMAN (PNEC freshwater /10)	
Imidacloprid (138261–41-3) [TT 604] [67]	L	Settl	Normal	111	105.2	96	0.00056	ECHA (PNEC freshwater /10)	
Irgarol 1051 (28159–98-0) [TT 606] [70]	U	Settl	Normal	**0.001**	–	96	0.0025	INERIS (AA-QS marine eco)	*
MEMC (123–88-6) [TT 169] [66]	U	Fert	Normal	1	–	3	Unknown		
Permethrin (52645–53-1) [TT 612] [66]	U	Settl	Normal	0.3	–	18	0.00002	NORMAN (PNEC freshwater /10)	
Profenofos (41198–08-7) [TT 620] [66]	U	Settl	Normal	0.1	–	18	0.0004	ECHA (PNEC freshwater /10)	
Propiconazole (60207–90-1) [TT 621] [67]	L	Settl	Normal	333	228.092	96	0.68	ECHA (PNEC marine water)	
Tributyltin (688–73-3) [TT 631] [60]	U	Settl	High T°	0.35	–	24	0.0002	INERIS (AA-QS marine eco)	
UV filter									
Benzophenone-1 (131–56-6) [TT 12] [71]	L	Bleach	Low pH	100	45.905	168	3.27	ECHA (PNEC freshwater /10)	
Benzophenone-2 (131–55-5) [TT 278] [72]	U	Mort	Normal	0.615	–	4	Unknown		
Benzophenone-3 (131–57-7) [TT 18] [73]	M	Bleach	Normal	2.28	–	8	0.067	ECHA (PNEC freshwater /10)	
Benzophenone-8 (131–53-3) [TT 556] [71]	L	Settl	Low pH	1	0.65	336	Unknown		
Detergent									
Linear alkylbenzene sulfonate (85536–14-7) [TT 342] [74]	L	Mort	Normal	750	750	24	26.8	ECHA (PNEC freshwater /10)	
Nonylphenol ethoxylate (9016–45-9) [TT 347] [74]	L	Mort	Normal	1000	1000	24	0.8	ECHA (PNEC freshwater and marine water, value for nonylphenol branched ethoxylated)	

Chemical name and CAS number, risk of bias (*L* low , *U* unclear, or *M* medium), outcome (*Fert* fertilisation, *Settl* settlement, *Bleach* symbiont density, *Photo* symbiont photosynthesis and *Mort* mortality), temperature and pH conditions of the studies used to determine the TT, lowest TT concentration value in µg/L (nominal and effective when measured) and exposure duration in hour, reference value used to contextualise the TT and source of the reference value. The term AA-QS means annual average quality standard. Reference values appear protective for corals for all the chemicals assessed except three that are highlighted with a star. With chemical name within square brackets is the index number given to each set of effect size estimates used to determine the toxicity threshold (see Additional file 5) and references of the corresponding primary research articles

##### Narrative synthesis of study findings

The 150 studies included in the narrative synthesis of the findings, which focuses on exposure categories Detergent, Dispersant, Microplastic, Nanoparticle, Pharmaceutical, UV filter, and Other (Table 4), were split into 447 cases, each case being a single concentration-duration tested within a study (Additional file 5, “Cases narrative synthesis” sheet). Of these, the overall risk of bias was defined as being low for 38 studies (76 study cases), unclear for 90 (291), medium for 14 (51), and high for 8 (29).

##### Detergent

In the detergent category, the exposures recorded in the narrative synthesis were also recorded in the quantitative synthesis, with the exception of exposure to 4-nonylphenol. Studies with a low risk of bias showed a statistically significant negative effect of linear alkylbenzene sulfonate on the horizontal tissue growth of *Sty. pistillata* and *Poc. damicornis* at concentrations of 0.75, 1 and 5 mg/L for 24 h, but no significant effects for nonylphenol ethoxylate [74] (Table 9). Studies with an unclear risk of bias showed no significant effect of 4-nonylphenol on several parameters such as the release of *Poc. damicornis* planulae, the content in cholesterol, the content in steroids (estrone, estradiol, testosterone and progesterone), and the activity of several enzymes such as the 3-beta-hydroxysteroid dehydrogenase, the cytochrome P450, the glutathione-S-transferase, and the beta-glucuronidase [75]. However, there was an increase in the activity of UDP-glycosyltransferase and a decrease in sulfotransferase 1A1 activity [75].

**Table 9:** Summary table of the findings detailed in the narrative synthesis (except the “Other category”)

Chemical	Exposure	Effect	Species	Bias
Detergent				
Linear alkylbenzene sulfonate [74]	0.75, 1 and 5 mg/L for 24 h	Decrease in horizontal tissue growth	*Stylophora pistillata, Pocillopora damicornis*	L
Nonylphenol ethoxylate [74]	0.1—5 mg/L for 24 h	[No effect on horizontal tissue growth]	*Stylophora pistillata, Pocillopora damicornis*	L
4-nonylphenol [75]	1 ppb for 42 days	[No effect on release of planula, cholesterol, estrone, estradiol, testosterone, progesterone, and 3-beta-hydroxysteroid dehydrogenase, cytochrome P450, glutathione-S-transferase, and beta-glucuronidase activity]	*Pocillopora damicornis*	U
4-nonylphenol [75]	1 ppb for 42 days	increase in UDP-Glycosyltransferase activity and decrease in sulfotransferase 1A1 activity	*Pocillopora damicornis*	U
Dispersant				
Corexit 9527 [76]	1 ppm for 24 h	[No effect on polyp retraction]	*Pseudodiploria strigosa*	L
Corexit 9527 [78]	1 ppm for 8 h	[no effect on incorporation of photosynthetic carbon in tissues]	*Pseudodiploria strigosa*	U
Corexit 9527 [79]	1–50 ppm for 8 h	[No effect on the gene expression of the heat shock protein Hsp90]	*Orbicella franksi*	U
Corexit 9527 [79]	10 and 50 ppm for 8 h	increase in the gene expression of P-glycoprotein	*Orbicella franksi*	U
Corexit 9527 [79]	5, 10 and 50 ppm for 8 h	increase in the gene expression of the heat shock protein Hsp70	*Orbicella franksi*	U
Corexit 9500 [77]	0.05% v/v for 13 days	[No effect on microbiome diversity]	*Millepora alcicornis*	U
Corexit 9500 [77]	0.05% v/v for 13 days	changes in microbiome structure	*Millepora alcicornis*	U
Oil-degrading bacteria consortium [77]	10^6 cells/mL for 13 days	[No effect on microbiome diversity and structure]	*Millepora alcicornis*	U
Corexit 9500 and oil-degrading bacteria consortium [77]	0.05% v/v & 10^6 cells/mL for 13 days	[No effect on microbiome diversity and structure]	*Millepora alcicornis*	U
Microplastic				
Artificial clothing fibers, automobile residues or beach microplastics [80]	10 mg/L for 10 weeks (+ heat stress)	[No effect on tissue bleaching and necrosis]	*Pocillopora verrucosa, Stylophora pistillata*	L
Polyethylene [80]	10 mg/L for 10 weeks (+ heat stress)	[No effect on tissue bleaching and necrosis]	*Acropora muricata, Montipora digitata, Pocillopora verrucosa, Porites cylindrica, Stylophora pistillata*	L
Polyethylene [81]	200 particles/L for 6 months (size 65–410 µm)	[No effect on tissue bleaching and necrosis]	*Acropora muricata, Heliopora coerulea, Porites lutea*	L
Polyethylene [81]	200 particles/L for 6 months (size 65–410 µm)	Increase in bleaching	*Pocillopora verrucosa*	L
Polyethylene [82]	4000 particles/L for 4 weeks (size 37–163 µm)	Increase in bleaching	*Acropora millepora*	L
Polyethylene [81]	200 particles/L for 6 months (size 65–410 µm)	[No effect on symbiont chlorophyll concentration]	*Acropora muricata, Heliopora coerulea, Porites lutea, Pocillopora verrucosa*	L
Polyethylene [83]	5 or 50 particles/L for 28 days (size 106–125 µm)	[No effect on symbiont chlorophyll concentration]	*Stylophora pistillata*	L
Polyethylene [84]	5, 15, 25, 50, 100 or 200 particles/L for 3 h (size 1 or 6 µm)	[No effect on embryo development]	*Acropora tenuis*	L
Polyethylene [85]	30 mg/L for 12 weeks (mix of 3 size classes 212–250 µm, 425–500 µm, 850–1000 µm)	Decrease in tissue growth	*Pseudodiploria clivosa, Acropora cervicornis*	L
Polyethylene [83]	50 particles/L for 28 days (size 106–125 µm)	Increase in non-photochemical quenching and changes in tissue polar metabolite composition	*Stylophora pistillata*	L
Polypropylene [84]	5, 15 or 50 particles/L for 3 h (size 0.5, 1 or 2 mm^2^)	[No effect on embryo development]	*Acropora tenuis*	L
Polystyrene [90]	1 – 1000 mg/L for 96 h	[No effect on catalase activity and melanin content]	*Porites porites*	U
Polystyrene [89]	50 mg/L for 24 h (size 1 µm)	[No effect on symbiont chlorophyll concentration]	*Pocillopora damicornis*	U
Polystyrene [89]	50 mg/L for 12 h (size 1 µm)	changes in the transcriptome profile	*Pocillopora damicornis*	U
Microfibres and polystyrene [120]	0.1 mg/L for 12 days (size 0.05–1 cm and 500–1000 µm)	[No effect on respiration rates]	*Acropora* sp.*, Seriatopora hystrix*	U
PVC [86, 87]	300 mg/L for 24 h (size 1–10 µm) or 1, 30 mg/L for 72 h	Metabolic changes	*Tubastrea aurea*	U
PA66, polyethylene, PET or polystyrene [87–89]	50 or 300 mg/L for 12, 24 or 96 h (various sizes)	Metabolic changes	*Acropora* sp.*, Tubastraea aurea, Pocillopora damicornis*	M, U
Nanoparticle				
Silver nanocolloids [91]	50 µg/L for 10 days	Decrease in primary polyp growth	*Acropora japonica*	L
MeO-PEG-b-PMOT [93]	2 mg/mL for 24 h	Changes in the proteome composition	*Acropora tenuis*	U
CdSe/ZnS quantum dots [94]	0.1 – 50 nM for 12 h	Changes in the transcriptome profile	*Stylophora pistillata*	U
Titanium dioxide nanoparticles [92]	0.1 or 10 mg/L for 17 days	[No effect on the expression of various genes]	*Orbicella faveolata*	M
Pharmaceutical				
Estradiol [95]	2300 ng/L for 21 days	Decrease in the number of egg-sperm bundles	*Montipora capitata*	L
Estradiol [95]	2300 ng/L for 21 days	[No effect on egg surface area, number of eggs per bundle]	*Montipora capitata*	L
Estradiol [96]	0.5 or 5 ng/mL for 5 days	[No effect on the gene expression of vitellogenin]	*Acropora tenuis*	U
Estrone [95]	2 ng/L for 8 weeks	[No effect on tissue protein content]	*Porites compressa*	U
Several pharmaceuticals (Hydrogen peroxide, L-5-hydroxytryptophan, Naloxone hydrochloride dihydrate, Serotonin acetate monohydrate) [97]	0.01 µM – 2 mM for 72 h	[No effect on spawning]	*Acropora cervicornis*	U
ε-caprolactone-p-coumaric acid copolymers [98]	one square cm for 10 days	[no effect on branchiness]	*Acropora muricata*	U
Verapamil [100]	0.05 µM for 4 days, 0.2 µM during 2 days then 5 µM during 2 days	Increase in respiration rate	*Pocillopora damicornis*	U
Ciprofloxacin and various antibiotics mix [102–106]	Various concentrations and durations	Changes in bacterial community structure, diversity, composition and activity	*Acropora cervicornis, Pseudodiploria strigosa, Porites astreoides, Fimbriaphyllia paradivisa, Pocillopora damicornis, Acropora muricata*	U
Antibiotics (ampicillin, streptomycin, ciprofloxacin, naladixic acid) [105]	between 0.1 mg/mL and 1 mg/L depending on the antibiotic for 12 h every night during five days	[No effect on tissue protein content]	*Pocillopora damicornis*	U
Antibiotics (ciprofloxacin) [98]	0.014% (w/v) in one square cm for 10 days	[No effect on branchiness]	*Acropora muricata*	U
Antibiotics (nalidixic acid, ampicillin and streptomycin) [104]	0.1 mg/mL for 48 h	[No effect on gene expression]	*Fimbriaphyllia paradivisa*	U
Amoxicillin [101]	1.6 g /colonies for 3 days	(*) healing of tissue lesions	*Montastraea cavernosa, Orbicella faveolata, Diploria labyrinthiformis, Pseudodiploria strigosa*	M
Verapamil [99]	100 µmol/L for 2 h	[No effect on the incorporation of aspartic acid]	*Stylophora pistillata*	M
Antibiotics (ampicillin, penicillin and streptomycin) [107]	50 µg/mL for 6 days	Decrease in mRNA expression of the yolk protein vitellogenin in cultured ovaries	*Fimbriaphyllia ancora*	H
UV filter				
Benzophenone-3 [121]	1 µg/L for 41 days (+ heat stress)	Changes in the microbiome beta diversity	*Stylophora pistillata*	L
Benzophenone-3 [108]	2 mg/L for 7 days	Changes in metabolomic profile	*Pocillopora damicornis*	U
Bis-ethylhexyloxyphenol methoxyphenyl triazine, diethylhexyl butamido triazone, diethylamino hydroxybenzoyl hexyl benzoate, ethylhexyl triazone, homosalate and methylene bis-benzotriazolyl tetramethylbutylphenol [108]	1000 µg/L for 7 days	[No effect on the metabolomic profile]	*Pocillopora damicornis*	U
Avobenzone [108]	1000 µg/L for 7 days	Changes in metabolomic profile	*Pocillopora damicornis*	U
Octisalate [108]	5 – 1000 µg/L for 7 days	Changes in metabolomic profile	*Pocillopora damicornis*	U
Octocrylene [109]	50 – 1000 µg/L for 7 days	Changes in metabolomic profile	*Pocillopora damicornis*	U
Titanium dioxide [110]	6.3 mg/L for 48 h	Increase in the number of damaged algal symbionts	*Acropora* spp.	U
Zinc oxide nanoparticles [110]	6.3 mg/L for 48 h	Increase in the number of damaged algal symbionts	*Acropora* spp.	U
Zinc oxide nanoparticles [111]	(50 – 200 µg/L for 24 h)	Changes in the membrane lipid profile	*Seriatopora caliendrum*	U
Sunscreens with titanium dioxide nanoparticles Eusolex TS as UV filter [112]	0.1 mg/L or 1 mg/L for 12 days	Decrease in respiration	*Seriatopora hystrix*	U
Sunscreens with titanium dioxide nanoparticles Eusolex TS as UV filter [112]	0.1 mg/L or 1 mg/L for 12 days	[No effect on respiration]	*Seriatopora hystrix, Porites cylindrica*	U
Sunscreens with titanium dioxide nanoparticles Eusolex TS as UV filter [112]	0.05 – 1 mg/L for 5 h	Increase in abnormal embryo development rate	*Acropora hyacinthus*	U
Sunscreens with titanium dioxide nanoparticles Eusolex TS as UV filter [112]	0.05 – 1 mg/L for 5 h	[No effect on abnormal embryo development rate]	*Acropora hyacinthus*	U
Sunscreens with titanium dioxide nanoparticles Eusolex TS as UV filter [112]	1 mg/L for 15 min	Decrease in sperm motility	*Acropora globiceps*	U
Sunscreens with titanium dioxide nanoparticles Eusolex TS as UV filter and Cellulose NanoCrystal [112]	1 mg/L for 15 min	[No effect on sperm motility]	*Acropora globiceps*	U
Sunscreen with titanium dioxide nanoparticles Eusolex T-Avo [112]	0.05 – 1 mg/L for 5 h	Increase in abnormal embryo development rate	*Acropora hyacinthus*	U
Sunscreen with titanium dioxide nanoparticles Eusolex T-Avo [112]	0.05 – 1 mg/L for 5 h	[No effect on abnormal embryo development rate]	*Acropora hyacinthus*	U
Sunscreen with titanium dioxide nanoparticles Eusolex T-Avo [112]	1 mg/L for 15 min	Decrease in sperm motility	*Acropora globiceps*	U
Benzophenone-2 [72]	24.6 µg/L – 246 mg/L for 8 h or 24 h	Increase in the number of DNA lesions and planulae deformation	*Stylophora pistillata*	M
Benzophenone3 [73]	22.8 µg/L – 228 mg/L for 8 h or 24 h	Increase in the number of DNA lesions and planulae deformation	*Stylophora pistillata*	M

Chemicals are ordered by category and overall risk of bias of the studies within a category (low (L), unclear (U), medium (M) or high (H)). It should be noted that some studies did not test the toxicity but the ability of some pharmaceuticals to heal corals (*). References of the corresponding primary research articles are given within square brackets with the chemical name

##### Dispersant

In the dispersant category, the exposures recorded in the narrative synthesis were also recorded in the quantitative synthesis, and only one study, which showed no statistically significant effect of Corexit 9527 on polyp retraction of *Pseudodiploria strigosa*, has a low risk of bias [76] (Table 9). The other studies, with an unclear risk of bias, showed no significant effect of Corexit 9500, neither of an oil-degrading bacterial consortium, or of their combination on the microbiome diversity of *Millepora alcicornis*, as well as on its bacterial community structure (except for Corexit 9500 only) [77]; and no significant effect of Corexit 9527 on the incorporation of photosynthetic carbon in the tissues of *P. strigosa* colonies [78] nor on the gene expression of the heat shock protein Hsp90 of *Orbicella franksi* [79]. However, for *O. franksi*, a statistically significant increase in the gene expression of the P-glycoprotein was observed at concentrations of 10 and 50 ppm and in the gene expression of the heat shock protein Hsp70 at concentrations of 5, 10 and 50 ppm [79]. Although an increase in gene expression of these two proteins is generally indicative of a general cellular stress response [79], such increase can be transient after exposure to the pollutant, and is not indicative of any cellular or physiological damage to the corals.

##### Microplastic

In the microplastic category, the exposures recorded in the narrative synthesis were also recorded in the quantitative synthesis, with the exception of polyvinyl chloride (PVC) particles. Studies with a low risk of bias reported exposure to various microplastics residues, and polyethylene and polypropylene particles (Table 9). Their results show no additional long-term effect of artificial clothing fibers, automobile residues, beach microplastics, and polyethylene particles, on coral tissue bleaching and necrosis following heat stress (10 mg/L for 10–11 weeks) [80]. Regarding long-term exposure to polyethylene particles, no statistically significant effect on tissue bleaching and necrosis was found for *A. muricata*, *Heliopora coerulea*, and *Porites lutea* but the effect was significant for *Poc. verrucosa* (200 particles/L (size 65–410 µm) for 6 months exposure) [81] as well as for *A. millepora* (4000 particles/L (size 37–163 µm) for 4 weeks) [82]. Also, no statistically significant effect was found on chlorophyll concentration in symbiont (five coral species, 5 or 50 particles/L (size 106–125 µm) for 28 days or 200 particles/L (size 65–410 µm) for 6 month exposure) [81, 83] or on *A. tenuis* embryo development (gametes exposure at 5–200 particles/L (size 1 or 6 µm) for 3 h) [84]. However, exposure to polyethylene particles decreased tissue growth of *Pseudodiploria clivosa* and *A. cervicornis* (30 mg/L (mix of 3 size classes 212–250 µm, 425–500 µm, 850–1000 µm) for 12 weeks) [85], increased non-photochemical quenching and modified the tissue polar metabolite composition of *Sty. pistillata* (50 particles/L (size 106–125 µm) for 28 days) [83]. Regarding short-term exposure to polypropylene particles, no statistically significant effect was found on the embryo development of *A. tenuis* (gametes exposure at 5, 15 or 50 particles/L (size 0.5, 1 or 2 mm^2^) for 3 h [84]. Overall, these studies on polyethylene suggest that the effects of this plastic compound on corals are species-specific, as well as concentration and duration dependent. While the physiology of coral symbionts does not seem to be affected by polyethylene particles, coral hosts seem to be more impacted, decreasing tissue growth and changing its metabolome.

Studies with an overall unclear or medium risk of bias reported short-term exposure to particles of PVC, polyamide 66 (PA66), polyethylene, polystyrene, and polyethylene terephthalate (PET) and microfibres. They show, for PVC, a statistically significant increase in oxidative stress (catalase (CAT) activity and lipid peroxidation (LPO)), a decrease in metallothionein (MT) content after 72 h at 1 mg/L; the same effects—except a decrease in LPO concentration, were observed after 72 h at 30 mg/L; at 300 mg/L (size 1–10 µm) for 24 h, a statistically significant decrease in anti-oxidant molecules (CAT, superoxide dismutase (SOD), glutathione (GSH)), in total antioxidant capacity as well as in the activity of alkaline phosphatase (AKP), pyruvate kinase (PK), and ATPases (Na–K-ATPase, Ca-ATPase, Mg-ATPase, Ca-Mg-ATPase) was observed for *Tubastraea aurea* [86, 87]. These changes in antioxidant activity of the corals are however not directly informative on the effects of PVC particles on coral physiology. Indeed, these changes can be transient, and it’s only when the stress induced by PVC exposure exceeds the antioxidant capacity of the corals that PVC can induce lasting physiological damages. Similarly, short-term exposure to particles of PA66, polyethylene, PET or polystyrene significantly changed the activity of several enzymes in the corals *Acropora* sp., *T. aurea*, or *Poc. damicornis* (50 or 300 mg/L (various sizes) for 12, 24 or 96 h) [87–89] and short-term exposure to polystyrene particles modified the transcriptome profile of *Poc. damicornis* (50 mg/L (size 1 µm) for 12 h) [89]. Finally, short-term exposure to polystyrene particles did not significantly change the CAT activity and melanin content of *Porites porites* (1–1000 mg/L for 96 h) [90] and the chlorophyll concentration in symbionts of *Poc. damicornis* (50 mg/L (size 1 µm) for 24 h) [89], and medium-term exposure to microfibres and polystyrene (0.1 mg/L (size 0.05–1 cm and 500–1000 µm) for 12 days) did not change the respiration rate of *Acropora* sp. and *Ser. hystrix*.

##### Nanoparticle

In the nanoparticle category, the exposures recorded in the narrative synthesis were also recorded in the quantitative synthesis, with the exception of CdSe/ZnS quantum dots. The studies on nanoparticle-containing sunscreens are reported below in the “UV filter” section. Only one study has an overall low risk of bias (Table 9), and it shows a statistically significant negative effect of exposure to 50 µg/L silver nanocolloids for 10 days on the growth of *A. japonica* [91]. A study with an overall medium risk of bias shows no significant effect of titanium dioxide nanoparticles on the expression of various genes (*Orbicella faveolata*, 0.1 or 10 mg/L for 17 days) [92]. However, studies with an overall unclear risk of bias show that a 24 h exposure to 2 g/L redox polymer MeO-PEG-b-PMOT with ROS scavengers changed the proteome composition of *A. tenuis* larvae [93] and a 12 h exposure to 0.1 – 50 nM CdSe/ZnS quantum dots changed the transcriptome profile of* Sty. pistillata* [94].

##### Pharmaceutical

In the pharmaceutical category, several exposures recorded in the narrative synthesis are not recorded in the quantitative synthesis. It should be noted that some studies did not test the toxicity but the ability of the pharmaceuticals to “heal” corals (Table 9). Only two studies have an overall low risk of bias, and they show a statistically significant reduction in the number of egg-sperm bundles after exposure to estradiol (2300 ng/L for 21 days) but the egg surface area and the number of eggs per bundle did not change [95]. Studies with an overall unclear risk of bias also show no effect of estradiol on the gene expression of *A. tenuis* vitellogenin, a protein component of coral egg yolk [96]. Also, studies showed no significant effect of estrone on the protein content of *Porites compressa* [95], of hydrogen peroxide, L-5-hydroxytryptophan, naloxone hydrochloride dihydrate, and serotonin acetate monohydrate on *A. cervicornis* spawning [97], and of ε-caprolactone-p-coumaric acid copolymers on the branchiness of *A. muricata* [98]. Studies with an overall unclear or medium risk of bias show that verapamil (a pharmacological inhibitor) did not alter the incorporation of aspartic acid into *Sty. pistillata* tissue and skeletal proteins [99], however it increased *Poc. damicornis* respiration rate after a 4 days exposure at 0.05 and 0.2 µM (but not at 1 µM) [100]. Regarding antibiotics, 19 studies with an overall unclear or medium risk of bias show that they could facilitate healing of tissue lesions (4 species) [101] and modify coral bacterial community structure, diversity, composition and activity (6 species) [102–106], but that they had no effect on *Poc. damicornis* tissue protein content [105], *A. muricata* branchiness [98] and *Fimbriaphyllia paradivisa* gene expression [104]. One study however found that antibiotics decreased mRNA expression of the yolk protein vitellogenin in *Fimbriaphyllia ancora* cultured ovaries but the study has a high risk of bias due to the presence of a confounding factor [107].

##### UV filter

For the UV filter category, several of the exposures recorded in the narrative synthesis are not recorded in the quantitative synthesis. Only two studies have an overall low risk of bias, and they show that benzophenone-3 changed the microbiome diversity of *Sty. pistillata* when combined with heat stress (Table 9). The other studies have an overall unclear or medium risk of bias. They show a statistically significant increase in the number of DNA lesions and proportion of abnormal shaped planulae (“deformation”) in *Sty. pistillata* (22.8 µg/L–228 mg/L for 8 h or 24 h) [73], and modification of the metabolomic profile of *Poc. damicornis* (2 mg/L for 7 days) [108], after benzophenone-3 exposure. Similarly, a statistically significant increase in the number of DNA lesions and proportion of deformation in *Sty. pistillata* planulae was found after benzophenone-2 exposure (24.6 µg/L – 246 mg/L for 8 h or 24 h) [72]. The metabolomic profile of *Poc. damicornis* was also significantly modified after exposure to avobenzone (1000 µg/L for 7 days), octisalate (5–1000 µg/L for 7 days) and octocrylene (50–1000 µg/L for 7 days) but not after exposure to bis-ethylhexyloxyphenol methoxyphenyl triazine, diethylhexyl butamido triazone, diethylamino hydroxybenzoyl hexyl benzoate, ethylhexyl triazone, homosalate, and methylene bis-benzotriazolyl tetramethylbutylphenol (1000 µg/L for 7 days) [108, 109]. Titanium dioxide nanoparticles Eusolex T2000 and Optisol significantly increased the number of damaged algal symbionts released by *Acropora* corals, as well as zinc oxide nanoparticles (6.3 mg/L for 48 h) [110]. Zinc oxide nanoparticles also modified the membrane lipid profile of *Seriatopora caliendrum* corals (50–200 µg/L for 24 h) [111]. Exposure to sunscreens with titanium dioxide nanoparticles Eusolex TS as UV filter had no effect or decreased the respiration rate of *Ser. hystrix*, but not of *Porites cylindrica* (0.1 mg/L–1 mg/L for 12 days) [112]. These sunscreens also had no effect or increased the abnormal development rate of *A. hyacinthus* embryo (0.1–1 mg/L for 5 h at normal or high temperature) and had no effect or reduced, depending on the other sunscreen components, *A. globiceps* sperm motility (1 mg/L for 15 min) [112]. Exposure to a sunscreen with titanium dioxide nanoparticles Eusolex T-Avo as UV filter had no effect or increased the abnormal development rate of *A. hyacinthus* embryo (0.05–1 mg/L for 5 h at normal or high temperature) and reduced *A. globiceps* sperm motility (1 mg/L for 15 min) [112].

##### Other chemicals

Finally, for chemicals that could not be classified elsewhere (Other category), exposures that were recorded in the narrative synthesis were also recorded in the quantitative synthesis. Only one study has an overall low risk of bias, and does not show a statistically significant effect of either hexabromocyclododecane (HBCDD, a flame retardant) or HBCDD containing polystyrene leachate on the respiration rate of *Sty. pistillata* (208–220 ng/L alpha-HBCDD, 16–25 ng/L beta-HBCDD, 2–8 ng/L gamma-HBCDD for 5 days) [113]. Studies with an overall unclear risk of bias show that ruthenium red (3.7–5.3 µM for 4 days) and glycolaldehyde (5 mmol/L for 3 h) respectively increased [100] and decreased [114] respiration rate of *Poc. damicornis* but that glycolaldehyde had no effect on the chlorophyll concentration in symbionts (3 mmol/L for 24 h at normal and high temperature) [114]. They also show that polychlorinated biphenyls (PCBs, the commercial mixture Aroclor1254) had no statistically significant effect on expression of several genes (293 ng/L for 4 h, heat shock protein 70-like, actin-related protein 2/3, ADP ribosylation factor 6-like, Rab7, glutaredoxin), and on horizontal growth of *Sty. pistillata* (293 ng/L for 96 h) [115]. However, 1,3,5-trinitro-1,3,5 triazine (a munition constituent) significantly modified *A. muricata* coral and algal symbiont transcriptomes (0.5–8 mg/L for 5 days) [116]. Several cryoprotectants also significantly reduced the size of *Poc. damicornis* tissue balls at high concentrations (3 – 4 M DMSO, 4 M ethylene glycol, 3–4 M glycerol, and 3–4 M methanol for 20 min) [117]. Studies with an overall high risk of bias show that cryoprotectants DMSO and propylene glycol decreased the number of mtDNA molecules in *Echinopora* oocytes (2–3 M for 20 min) but that cryoprotectants ethylene glycol, glycerol and methanol had no effect (0.5–3 M for 20 min) [118]. They also show that a vitrification solution with erucic acid increased the vitality of *Seriatopora caliendrum* larvae subjected to ultra-fast freezing (vitrification) (400 µg/µL for 4 min) followed by laser thawing, but not of *Poc. verrucosa* larvae, and the effect was not found when vitrification solutions contained linoleic acid, phosphatidylcholine, or phosphatidylethanolamine [119].

## Review limitations

### Limitations of the review methods

Due to limited resources, the screening, critical appraisal and data extraction steps were not carried out independently by two reviewers for all articles/studies. A careful check of the consistency of decisions showed good agreement, and clarified decision making where necessary. We therefore consider it unlikely that this would have substantially affected our conclusions.

The main limitation of the review methods is that a considerable number of studies (487 studies, 36%) were excluded from further synthesis because it was not possible to extract a valid result (no mean and/or sample size for the quantitative synthesis, no statistical tests for the narrative synthesis). These missing studies mainly concern the mortality outcome (180 studies), and the categories UV filter (108 studies) and Pesticide (100 studies).

Another limitation is that the data extraction method did not take into account a possible delayed response to chemical exposure. Indeed, in the data extraction, we considered the result obtained at the closest possible time point after the end of exposure to avoid taking into account a possible recovery after exposure.

In addition, the narrative synthesis of findings summarises the results of statistical tests but the validity of the statistical approaches was not assessed. Errors in statistical methods applied within individual studies has been recently highlighted as a source of bias that should be considered in environmental systematic reviews [122].

Finally, it should be noted that in the quantitative synthesis different proxies for the outcome categories “growth”, “symbiont density (bleaching)”, and “photosynthetic performance” were measured in the studies, with some being more relevant than others (see section “Data coding and extraction strategy”). When multiple proxies were present in the same study, the most relevant one was always selected. All proxies were included in our analysis within an outcome category, and the potential heterogeneity in effect size estimates that might result was not assessed. However, details of the variables measured for the outcome categories are provided in Additional file 5.

### Limitations of the evidence base

Because the majority (95%) of the toxicity thresholds identified by chemical, outcome, species and life stage were each obtained from a single article, the risk of publication bias and the influence of individual studies on the results are very high. This strong influence of individual studies on the results is problematic because laboratory conditions and coral origin or genotype, as well as an association with different algal symbionts and other microorganisms may have influenced the coral response to chemicals [123–125].

This review also shows that the ecotoxicological information is available for only few coral species compared to the wide diversity of corals, and experiments are needed with corals from less studied geographical regions or functional groups. In particular, most studies dealt with fast-growing branching corals (*Stylophora pistillata*, *Pocillopora damicornis*, *Acropora* spp.), because they can be easily broken down into small nubbins, and therefore, can be easily used in experiments with multiple replication of chemical concentrations and sampling times. Massive slow-growing corals, such as *Porites* species, have been much less studied. These corals tend to be associated with distinct microbial communities and are considered more resilient to environmental stressors such as seawater warming, and thus may respond differently to chemicals [126–128]. In addition, the second most studied species *Poc. damicornis* is now redescribed as a species complex. When several studies were used to determine the toxicity threshold, the non-monotonic nature of some dose–response relationships might be explained by species differences. However, the bias is likely limited, as the majority of toxicity thresholds are based on the same study and the origin of corals within a study is usually the same. Finally, the coral holobiont has never been considered in its entirety. Indeed, studies have examined the effects of pollutants on either the coral host, the dinoflagellate symbionts, or the other microbial communities, but very few studies have considered the parallel response of the various components of the holobiont to a contaminant. Therefore, a lack of significant effect of a pollutant on the dinoflagellate symbionts does not exclude a deleterious effect of that pollutant on the other partners.

Another limitation is that more than half of the studies (55.5%) did not measure or report effective exposure concentrations, but only referred to nominal concentrations. Given the large differences observed for some substances between nominal and effective exposure concentrations (see Fig. 9), this represents a severe limitation. This could be particularly problematic for the category of hydrocarbons, where effective concentrations are likely to vary according to the preparation method (e.g. water accommodated fraction, water soluble fraction, O-rings). This could also be problematic for organic UV filters, as it has been shown that organic UV filters are hydrophobic substances that adhere to beaker and aquarium surfaces and are then no longer dissolved in seawater and available to corals [46]. In addition, most studies have been conducted using high or even very high concentrations of chemicals, compared to concentrations normally measured in seawater, and with exposure times much shorter (a few hours to days at most) than the in situ timescales during which most corals are actually exposed to environmental pollutions (which may be continuous exposure to infinitesimal amounts of chemicals). For example, concentrations of UV filters measured in waters around coral reefs have been documented mostly in the ng/L (ppt) range [129–131] (but see [73]), but UV filters have been tested experimentally in aquaria at concentrations at least 10 times higher (see Additional file 5). Even if such high concentrations applied for only a few hours to days prove not to be harmful to corals, this does not preclude lower concentrations applied over a longer period of time from exerting chronic toxicity. Therefore, because most studies on coral response to contaminants have been conducted in short-term, small-scale laboratory experiments at the organism level, it is difficult to extrapolate the results obtained here to determine how contaminants may alter in situ coral communities at ecologically more realistic temporal and spatial scales.

Finally, another limitation is that the toxicity tests have mostly been performed on corals with a single chemical, whereas most contaminants occur as a mixture of chemicals in the environment. This is true for pharmaceuticals, among others, but also for pesticides, herbicides, metals, and UV filters [132–134]. Although the contamination of surface waters with multiple pollutants is sometimes well documented through ongoing monitoring, little is known about their combined effects on corals, which can be antagonistic, synergistic or additive [34]. The current approach to ecological risk assessment therefore ignores the combined effects of multiple pollutants and likely underestimates true toxicity. Moreover, in this era of environmental changes, it is also of prime importance to take into account the combined effects of local stressors (pollutants) and global stressors (e.g. seawater warming and acidification) on corals. Several studies have indeed found that the threat to coral reefs posed by climate change may be further exacerbated by elevated levels of nutrients, sediments, and pollutants, brought to the corals colonies and their larvae by river discharge [23, 50, 135–137]. However, the effects of these stressors may or may not be synergistic. For example, the combination of thermal stress and Cu loading did not affect the primary production of symbionts associated with *Porites cylindrica* [138], but they acted synergistically in altering the metabolism of the host coral *Mussismilia hartii* [139]. Therefore, future studies should aim at investigating which contaminants act synergistically with global change stressors and further reduce the coral capacity to resist global change.

## Review conclusions

This systematic review provides an open-access database on the known ecotoxicological effects of chemical exposures on corals. The quantitative synthesis records the effects of 2706 exposure concentrations-durations of 164 chemicals or mixtures of chemicals, and identifies 105 toxicity thresholds corresponding to 56 chemicals or mixtures of chemicals.

### Implications for policy/management

The database provided by this systematic review can assist managers in the ecological risk assessment of chemical pollutants, by facilitating the determination of various ecotoxicological thresholds (e.g. the No Observed Effect Concentration (NOEC) or the Lowest Observed Effect Concentration (LOEC)). It also contains key information to contextualise the results such as the experimental conditions (experimental system, pH, temperature), the life stage of the tested species, the effective exposure concentration (if measured), and the risk of bias of the study. It should be noted, however, that the critical appraisal grid used here does not include some of the criteria used in regulatory risk assessment (e.g. the presence of positive control). Therefore, some studies qualified here as having an overall low risk of bias would be considered unusable under other grids (e.g. CRED: Criteria for Reporting and Evaluating Ecotoxicity Data. [140]).

Considering the limitations listed in the previous section for the toxicity tests reviewed, it can be concluded that most of the currently available data on coral toxicity needs substantial further development. In addition, organizations involved in reef policy and management have to keep in mind that, at the organism level, ecotoxicological studies on corals are challenging because corals are diverse and complex organisms that include an animal, microalgae, and associated microorganisms. And currently, there are no standardized tests implemented for corals, as there are for some freshwater organism models. At the ecosystem level, land-based pollution is one of the most important local stressors contributing to the loss of coastal coral reefs. Identifying potential pollutants is key for reef conservation, but it is also a major challenge to determine the cause-and-effect relationships between coral response and pollutant exposure over long timescales, and the relative contributions of co-occurring multiple pollutants. There are also top-down effects of pollutants on corals, as highlighted in a recent review [141]. For example, pollutants can indirectly favour macroalgae development at the expense of coral growth, and algal turfs significantly reduce coral recruitment [142]. Fish stocks are also important to coral health [143], and any decline in fish stocks due to seawater pollution can also affect the entire reef ecosystem. Therefore, the risk of any type of pollution should also be considered at the ecosystem level rather than just at the organism level.

### Implications for research

This database will help researchers identify the knowledge gaps related to the species, chemical pollutants, and outcomes studied. Since most of the identified toxicity thresholds are based on a single study, this will also help to replicate those studies to confirm the results. By knowing all exposure concentrations that have already been tested, this database also allows researchers to easily determine the concentration ranges to test.

To allow comparison between studies, standardized toxicity tests for corals are urgently needed [144]. In particular, it is imperative to determine the health status of the corals at the beginning of the experiments and to provide a complete description of the culture parameters (light, temperature, current, etc.) used during the experiments. Even if a control was conducted in parallel with the toxicity tests, experiments performed with healthy corals may not yield the same results as when conducted with unhealthy animals under less than optimal conditions. The most important aspects to consider in standardized toxicity tests were listed in a recent review paper [145] and should be taken into account.

## Supplementary Information

**Additional file 1**. ROSES systematic review checklist. ROSES form for systematic review version 1.0.

**Additional file 2.** Details for the search update. Details of all the searches for the update of literature with dates of search and number of articles found.

**Additional file 3.** List of excluded articles and studies with reasons for exclusion. List of articles excluded at full text screening when updating cluster 4 of the systematic map; list of studies excluded from cluster 4 of the systematic map; list of studies excluded from further synthesis with reasons.

**Additional file 4.** Critical appraisal. Critical appraisal definition and results for the 1348 studies included in the narrative synthesis of the characteristics of studies.

**Additional file 5.** Systematic review databases. List of articles included in quantitative synthesis and/or narrative synthesis of the findings; list of study cases of the quantitative synthesis; list of toxicity thresholds; list of study cases of the narrative synthesis of the findings.

## Data Availability

All data generated or analysed during this study are included in this published article and its Additional files.
