# Supplementary material for: What are the toxicity thresholds of chemical pollutants for tropical reef-building corals? A systematic review
Source: Environ Evid. 2023 Mar 19;12:4. doi: 10.1186/s13750-023-00298-y (PMC11378836; doi:10.1186/s13750-023-00298-y)
Supplement: Supplementary file 1 — Additional file 1. ROSES systematic review checklist. ROSES form for systematic review version 1.0. [file 13750_2023_298_MOESM1_ESM.pdf]

| Section/sub-section | Topic | Description | Further explanation | Checklist/meta-data | Author response | Comments |
|---|---|---|---|---|---|---|
| Title | Title | The title must indicate that it is a systematic review, and should indicate if it is an update/amendment: e.g. "…A systematic review update." | The title should normally be the same or very similar to the review question. | Meta-data | What are the toxicity thresholds of chemical pollutants for tropical reef-building corals? A systematic review | |
| Type of review | Type of review | Select one of the following types of review: systematic review, systematic review update, systematic review amendment, systematic review from a systematic map | See CEE Guidance on amendments and updates [1] | Meta-data | systematic review from a systematic map | |
| Authors' contacts | Authors' contacts | The full names, institutional addresses and email addresses for all authors must be provided. | | Checklist | yes | |
| Abstract | Structured summary | The abstract of the manuscript must not exceed 500 words and must be structured into separate sections: Background, the context and purpose of the review, including the review question; Methods, how the review was performed and statistical tests used (specifically mention search strategy, inclusion criteria, critical appraisal, data extraction and synthesis); Results, the main findings, including results of search and assessment of evidence base; Conclusions, brief summary and potential implications for policy/management and research. | | Checklist | yes | |
| Background | Background | Describe the rationale for the review in the context of what is already known. Reviews must indicate why this study was necessary and what it aims to contribute to the field. | A theory of change and/or conceptual model should be presented that links the intervention or exposure to the outcome. | Checklist | yes | |
| Stakeholder engagement | Stakeholder engagement | The actual role of stakeholders throughout the review process (e.g. in the formulation of the question) must be described and explained (using a broad definition of 'stakeholder', including e.g. researchers, funders and other decision-makers; see [2]) | | Checklist | yes | |
| Objective of the review | Objective | Describe the primary question and secondary questions (when applicable). | The primary question is the main question of the review. The secondary questions are usually linked to sources of heterogeneity (effect modifiers). | Checklist | yes | |
| | Definition of the question components | Provide reference to the question key elements, e.g. population(s), intervention(s)/exposure(s), comparator(s), and outcome(s). | For other question types see [3,4] | Meta-data | Population: all tropical reef-building coral species (hermatypic scleractinian species, Millepora species, Heliopora species and Tubipora species). All developmental stages are considered (mobile planula, fixed polyp), as well as all coral compartments including dinoflagellate symbionts "in hospite" and the microbiome.<br>Exposure: all geogenic (e.g. trace metals) and synthetic chemicals (e.g. diuron) for which exposure concentrations are known. Inorganic and organic dissolved nutrients (e.g. nitrate) are excluded.<br>Comparator: population not exposed to chemicals; Population before chemical exposure.<br>Outcome: all outcomes related to the health status of tropical reef-building corals, from molecular level (e.g. gene expression, enzyme activities) to colony (e.g. photosynthesis, bleaching) and population level (e.g. mortality rate).<br>Type of study: all experimental studies i.e. where exposure is controlled by researchers, in the laboratory or in the field. | |
| Methods | Protocol | Provide citation, DOI or open-access link to published protocol. | The protocol should be peer-reviewed and publicly available online (open access). | Meta-data | https://doi.org/10.1186/s13750-021-00250-y | |
| | Deviations from protocol | Describe any ways in which the final methods of the review deviate from those set out in the protocol along with a justification. | | Checklist | yes | |
| Searches | Search strategy | Detail the search strategy used, including: database names accessed, dates of searching, institutional subscriptions (or date ranges subscribed for each database), search options (e.g. 'topic words' or 'full text' search facility), efforts to source grey literature, other sources of evidence (e.g. hand searching, calls for evidence/submission of evidence by stakeholders). | | Checklist | yes | |
| | Search string | Provide Boolean-style full search string and state the platform for which the string is formatted (e.g. Web of Science format) | | Meta-data | The search string for the search update is as follows (Web Of Science format):<br>TS=(coral$ AND (toxicant$ OR chemical$ OR biocide$ OR "industrial product$" OR "consumer product$" OR "household product$" OR "biocidal product$" OR disinfect* OR oil OR metal$ OR pesticide$ OR herbicide$ OR insecticide$ OR fungicide$ OR antifoul* OR anti-foul* OR organochlorine$ OR "flame retardant$" OR detergent$ OR "perfluorinated compound$" OR pharmaceutical$ OR "personal care product$" OR cosmetic$ OR PAH$ OR petroleum OR hydrocarbon$ OR microplastic$ OR nanoparticle$ OR nano-particle$ OR "endocrine disrupt*" OR "organic compound$" OR dispersant$ OR metalloid$ OR solvent$ OR petrochemical$ OR additive$ OR preservative$ OR plasticizer$ OR hormone$ OR "transformation product$" OR "degradation product$" OR byproduct$ OR by-product$ OR sunscreen$ OR "UV filter$" OR "ultraviolet filter$" OR antibiotic$ OR phthalate$ OR PCB$ OR cyanide$ OR chlordecone OR nickel OR copper OR zinc OR cadmium OR mercury OR iron)) | |
| | Languages - bibliographic databases | List languages used in bibliographic database searches | | Meta-data | English | |
| | Languages – grey literature | List languages used in organisational website searches and web-based search engines | | Meta-data | English, French | |
| | Bibliographic databases | Provide the number of bibliographic databases searched | | Meta-data | 2 | |
| | Web-based search engines | Provide the number of web-based search engines searched | | Meta-data | 3 | |
| | Organisational websites | Provide the number of organisational websites searched | | Meta-data | 11 | |
| | Estimating comprehensiveness of the search | Describe the process by which the comprehensiveness of the search strategy was assessed (i.e. list of benchmark articles) | | Checklist | no | Comprehensiveness of the search assessed for the systematic map (https://doi.org/10.1186/s13750-021-00237-9) |
| | Search update | Describe any update to searches undertaken during the conduct of the review | Compulsory (if update performed). A search update is good practice if original searches were performed more than two years prior to review completion. | Checklist | yes | |
| Article screening and study inclusion criteria | Screening strategy | Describe the methodology for screening articles/studies for relevance. Methods for consistency of screening decisions (at title, abstract, and full texts levels) checking must be described. | | Checklist | yes | |
| | Inclusion criteria | Describe the inclusion criteria used to assess relevance of identified articles/studies. These must be broken down into the question key elements (e.g. relevant subject(s), intervention(s)/exposure(s), comparator(s), outcome(s), study design(s)) and any other restrictions (e.g. date ranges or languages). | | Checklist | yes | |
| Critical appraisal | Critical appraisal strategy | Describe here the method used for critical appraisal of study validity (including assessment of individual studies and the evidence base as a whole). Describe how repeatability of critical appraisal of study validity was tested. | | Checklist | yes | |
| | Critical appraisal used in synthesis | Describe how the information from critical appraisal was used in synthesis. | | Checklist | yes | |
| Data extraction | Meta-data extraction and coding strategy | Describe the method for meta-data extraction and coding for studies, providing lists of variables that will be extracted as meta-data and those that will be coded. Describe how repeatability of meta-data/data extraction and coding was tested. | Optional, a map database can be included within a systematic review | Checklist | n/a | |
| | Data extraction strategy | Describe the method for extraction of qualitative and/or quantitative study findings. Describe how repeatability of data extraction was tested. | | Checklist | yes | |
| | Approaches to missing data | Describe any process for obtaining and confirming missing or unclear information or data from authors. | | Checklist | yes | |

| | | | | | |
|---|---|---|---|---|---|
| Potential effect modifiers/reasons for heterogeneity | Potential effect modifiers/reasons for heterogeneity | Provide a list of and justification for the effect modifiers/reasons for heterogeneity that will be considered in the review. Also provide details of how the list was compiled (including consultation of external experts). | | Checklist | yes |
| Data synthesis and presentation | Type of synthesis | State the type of synthesis conducted as part of the systematic review (narrative only, narrative and quantitative, narrative and qualitative, narrative, qualitative and quantitative, narrative and mixed-methods) | | Meta-data | narrative and quantitative |
| | Narrative synthesis strategy | Describe methods used for narratively synthesising the evidence base in the form of descriptive statistics, tables (including SM database) and figures. Study findings must only be narratively synthesised and vote-counting must be avoided. | | Checklist | yes |
| | Quantitative synthesis strategy | If data are appropriate for quantitative synthesis, describe methods for calculating effect sizes, methods for handling complex data, statistical methods for combining data from individual studies, and any exploration of heterogeneity and publication bias. If all studies were not selected for synthesis explain criteria for selection (e.g. incomplete or missing information). | Compulsory (if quantitative synthesis performed) | Checklist | yes |
| | Qualitative synthesis strategy | Describe methods used for synthesising qualitative data and justify your methodological choices. Describe if and how you plan to analyse subgroups/subsets of data. If all studies were not selected for synthesis explain criteria for selection (e.g. incomplete or missing information). | Compulsory (if qualitative synthesis performed) | Checklist | n/a |
| | Other synthesis strategies | Describe any other approaches used for synthesising data or combining qualitative and quantitative syntheses (e.g. mixed methods) and justify your choice of methodology. | Compulsory (if other synthesis performed) | Checklist | n/a |
| | Assessment of risk of publication bias | Describe methods for examining the possible influence of publication bias on the synthesis. | This may be done for quantitative syntheses using diagnostic plots or statistical tests. | Checklist | yes |
| | Knowledge gap and cluster identification strategy | Describe the methods used to identify and/or prioritise key knowledge gaps (unrepresented or underrepresented subtopics that warrant further primary research) and knowledge clusters (well-represented subtopics that are amenable to full synthesis via systematic review). | Optional | Checklist | n/a |
| | Demonstrating procedural independence | Describe the role of systematic reviewers (who have also authored articles to be considered within the review) in decisions regarding inclusion or critical appraisal of their own work. | Reviewers who have authored articles to be considered within the review should be prevented from unduly influencing inclusion decisions, for example by delegating tasks appropriately. | Checklist | yes |
| Results | Description of review process | Describe the review process including the volume of evidence identified from all sources and retained through each stage of the review. Must also display the number of articles/studies included at all stages of the review in a flow diagram, including the number of articles/studies excluded at each stage. | | Checklist | yes |
| | Number of search results | Provide the number of search results from bibliographic databases (including updates if conducted) prior to duplicate removal. | This number should not include web-based search engine or organisational website searches: this will help assessment of the efficiency of the primary search string. | Meta-data | 2435 |
| | Number of search results after duplicate removal | Provide the total number of search results from bibliographic database searches following duplicate removal. | This number should not include web-based search engine or organisational website searches: this will help assessment of the efficiency of the primary search string. | Meta-data | 1314 |
| | Full text screening excludes | Additional file containing list of and reasons for full text exclusions. | | Checklist | yes |
| | Title screening results | Provide the number of articles retained following title screening. | Optional if screening titles and abstracts together | Meta-data | n/a |
| | Abstract screening results | Provide the number of articles retained following abstract screening. | Optional if screening titles and abstracts together | Meta-data | n/a |
| | Title and abstract screening results | Provide the number of articles retained following title and abstract screening. | Optional if screening titles and abstracts separately | Meta-data | 272 |
| | Retrieval results | Provide the number of articles retrieved at full text. | | Meta-data | 253 |
| | Unobtainable articles | Additional file containing list of unobtainable articles. | | Checklist | yes |
| | Full text screening results | Provide the number of articles retained following full text screening. | | Meta-data | 40 |
| | Consistency checking: screening | Results of consistency checking at all stages (screening, data extraction, critical appraisal) must be provided. Provide the number of titles, abstracts and full texts screened and checked for consistency by two or more reviewers as a fraction of the total (e.g. Title: 2000/20000; Abstract: 500/5000: Full text: 10/100). | | Checklist | yes |
| | Critical appraisal exclusions | If any studies are excluded due to low validity, provide the number of studies excluded from further synthesis during critical appraisal. | Compulsory for any studies not included in synthesis due to validity. Reviews authors may prefer to perform a sensitivity analysis (repeating analyses to examine the influence of validity) rather than excluding studies from synthesis. | Meta-data | 0 |
| | Narrative synthesis | Describe the body of evidence identified using figures and tables, avoiding vote-counting (tallying of studies based on results; direction or significance). Each must be presented with descriptive information (meta-data) and extracted study findings. Describe the validity of individual studies and the evidence base as a whole. | | Checklist | yes |
| | Extracted data | Additional file containing extracted quantitative or qualitative data (study findings) from included studies. | | Checklist | yes |
| | Systematic map database | Additional file containing meta-data and coding for included studies. | Optional, a map database can be included within a systematic review | Checklist | n/a |
| | Quantitative synthesis | Present results of quantitative synthesis of study findings (e.g. meta-analysis). | Compulsory (if quantitative synthesis performed) | Checklist | yes |
| | Qualitative synthesis | Present results of qualitative analysis of study findings (e.g. summaries of identified themes or categories). Also provide additional file with the identified themes or categories for each study. | Compulsory (if qualitative synthesis performed) | Checklist | n/a |
| | Other synthesis | Present results of any other synthesis methods used. | Compulsory (if other synthesis performed) | Checklist | n/a |
| | Risk of publication bias | Describe the results of assessments for the possible influence of publication bias on the synthesis. | For quantitative syntheses this may be done using diagnostic plots or statistical tests | Checklist | yes |
| Discussion | Discussion | Discuss the review results and suggest further enquiry or analysis (e.g. potential reasons for heterogeneity in outcome). Authors may draw attention to specific knowledge gaps. | | Checklist | yes |
| | Limitations of the review | Discuss possible limitations in the methods used. | | Checklist | yes |
| | Limitations of the evidence base | Discuss possible limitations in the evidence base. | | Checklist | yes |
| Conclusions | Implications for policy/management | Summarise the state of the evidence base and discuss the way in which the identified evidence may inform policy/practice decision making in relation to the review question. Provide any measure of the uncertainty surrounding the outcome. | Reviews must not include practical environmental management recommendations or advocacy. | Checklist | yes |
| | Implications for research | Discuss the way in which the identified evidence may inform research including options for increasing the reliability of study design that could improve future research. | In this section some advocacy for future research on the reviewed topic is permissible provided it is clearly justified by the review outcome/critical appraisal of study validity. | Checklist | yes |
| Declarations | Competing interests | Describe of any financial or non-financial competing interests that the review authors may have. | | Checklist | yes |

**References**


[1] Bayliss, H.R., Haddaway, N.R., Eales, J., Frampton, G.K. and James, K.L., 2016. Updating and amending systematic reviews and systematic maps in environmental management. Environmental Evidence, 5(1), p.20.

[2] Haddaway, N.R., Kohl, C., da Silva, N.R., Schiemann, J., Spök, A., Stewart, R., Sweet, J.B. and Wilhelm, R., 2017. A framework for stakeholder engagement during systematic reviews and maps in environmental management. *Environmental Evidence* , *6* (1), p.11.

[3] Collaboration for Environmental Evidence. 2018. Guidelines and Standards for Evidence synthesis in Environmental Management. Version 5.0. www.environmentalevidence.org/information-for-authors.

[4] Leeds Institute of Health Sciences. https://medhealth.leeds.ac.uk/info/639/information_specialists/1500/search_concept_tools. Accessed 12/11/2017.
