# Supplementary material for: What are the toxicity thresholds of chemical pollutants for tropical reef-building corals? A systematic review
Source: Environ Evid. 2023 Mar 19;12:4. doi: 10.1186/s13750-023-00298-y (PMC11378836; doi:10.1186/s13750-023-00298-y)
Supplement: Supplementary file 2 — Additional file 2. Details for the search update. Details of all the searches for the update of literature with dates of search and number of articles found. [file 13750_2023_298_MOESM2_ESM.docx]

**Additional file 2: Details for the search update.** Details of all the searches for the update of literature with dates of search and number of articles found.

| **Search performed** | **Date of search** | **Number of records** |
| --- | --- | --- |
| Publication database – **Scopus (Elsevier)**  TITLE-ABS-KEY ( coral AND ( toxicant OR chemical OR biocide OR "industrial product" OR "consumer product" OR "household product" OR "biocidal product" OR disinfect* OR oil OR metal OR pesticide OR herbicide OR insecticide OR fungicide OR antifoul* OR anti-foul* OR organochlorine OR "flame retardant" OR detergent OR "perfluorinated compound" OR pharmaceutical OR "personal care product" OR cosmetic OR pah OR petroleum OR hydrocarbon OR microplastic OR nanoparticle OR nano-particle OR "endocrine disrupt*" OR "organic compound" OR dispersant OR metalloid OR solvent OR petrochemical OR additive OR preservative OR plasticizer OR hormone OR "transformation product" OR "degradation product" OR byproduct OR by-product OR sunscreen OR "UV filter" OR "ultraviolet filter" OR phthalate OR pcb OR cyanide OR chlordecone OR antibiotic OR nickel OR copper OR zinc OR cadmium OR mercury OR iron ) ) AND PUBYEAR > 2019 | January 3^rd^ 2022 | 1336 |
| Publication database - **Web of Science Core Collection (Clarivate Analytics)**  TS=(coral$ AND (toxicant$ OR chemical$ OR biocide$ OR "industrial product$" OR "consumer product$" OR "household product$" OR "biocidal product$" OR disinfect* OR oil OR metal$ OR pesticide$ OR herbicide$ OR insecticide$ OR fungicide$ OR antifoul* OR anti-foul* OR organochlorine$ OR "flame retardant$" OR detergent$ OR "perfluorinated compound$" OR pharmaceutical$ OR "personal care product$" OR cosmetic$ OR PAH$ OR petroleum OR hydrocarbon$ OR microplastic$ OR nanoparticle$ OR nano-particle$ OR "endocrine disrupt*" OR "organic compound$" OR dispersant$ OR metalloid$ OR solvent$ OR petrochemical$ OR additive$ OR preservative$ OR plasticizer$ OR hormone$ OR "transformation product$" OR "degradation product$" OR byproduct$ OR by-product$ OR sunscreen$ OR "UV filter$" OR "ultraviolet filter$" OR antibiotic$ OR phthalate$ OR PCB$ OR cyanide$ OR chlordecone OR nickel OR copper OR zinc OR cadmium OR mercury OR iron) )  Timespan=2020-2022 | January 3^rd^ 2022 | 1099 |
| Search engine - **CORE**  title:((coral OR corals) AND (toxicant OR toxicants OR chemical OR chemicals OR biocide OR biocides OR "industrial product" OR "industrial products" OR "consumer product" OR "consumer products" OR "household product" OR "household products" OR "biocidal product" OR "biocidal products" OR disinfect* OR oil OR metal OR metals OR pesticide OR pesticides OR herbicide OR herbicides OR insecticide OR insecticides OR fungicide OR fungicides OR antifoul* OR anti-foul* OR organochlorine OR organochlorines OR "flame retardant" OR "flame retardants" OR detergent OR detergents OR "perfluorinated compound" OR "perfluorinated compounds" OR pharmaceutical OR pharmaceuticals OR "personal care product" OR "personal care products" OR cosmetic OR cosmetics OR PAH OR PAHs OR petroleum OR hydrocarbon OR hydrocarbons OR microplastic OR microplastics OR nanoparticle OR nanoparticles OR nano-particle OR nano-particles OR "endocrine disrupt*" OR "organic compound" OR "organic compounds" OR dispersant OR dispersants OR metalloid OR metalloids OR solvent OR solvents OR petrochemical OR petrochemicals OR additive OR additives OR preservative OR preservatives OR plasticizer OR plasticizers OR hormone OR hormones OR "transformation product" OR "transformation products" OR "degradation product" OR "degradation products" OR byproduct OR byproducts OR by-product OR by-products OR sunscreen OR sunscreens OR "UV filter" OR "UV filters" OR "ultraviolet filter" OR "ultraviolet filters" OR phthalate OR phthalates OR PCB OR PCBs OR cyanide OR cyanides OR chlordecone OR antibiotic OR antibiotics OR nickel OR copper OR zinc OR cadmium OR mercury OR iron)) AND year:[2020 TO 2022] | January 3^rd^ 2022 | 178 |
| Search engine - **Google Scholar**  Search with the software “Publish or Perish” (version 7.15.2643.7260, https:// harzing.com/resources/ publish-or-perish, version accessed 16 March 2020). Search on title only (equations copy/paste in “title words” box). Timespan 2020-2022  S1: coral OR corals AND toxicant OR toxicants OR chemical OR chemicals OR biocide OR biocides OR disinfection OR disinfectant OR disinfectants OR oil OR metal OR metals OR pesticide OR pesticides OR herbicide OR herbicides OR insecticides OR insecticides  (122 results)  S2: coral OR corals AND fungicide OR fungicides OR antifoulant OR antifoulants OR antifouling OR organochlorine OR organochlorines OR detergent OR detergents OR pharmaceutical OR pharmaceuticals OR cosmetic OR cosmetics OR PAH OR PAHs OR petroleum  (5 results)  S3: coral OR corals AND hydrocarbon OR hydrocarbons OR microplastic OR microplastics OR nanoparticle OR nanoparticles OR dispersant OR dispersants OR metalloid OR metalloids OR solvent OR solvents OR petrochemical OR petrochemicals OR additive OR additives  (66 results)  S4: coral OR corals AND preservative OR preservatives OR plasticizer OR plasticizers OR hormone OR hormones OR byproduct OR byproducts OR sunscreen OR sunscreens OR phthalate OR phthalates OR PCB OR PCBs OR cyanide OR cyanides OR chlordecone  (14 results)  S5: coral OR corals AND antibiotic OR antibiotics OR nickel OR copper OR zinc OR cadmium OR mercury OR iron  (31 results) | January 3^rd^ 2022 | 238 |
| Search engine - **GreenFile**  Search on title only. Timespan 2020-2022  (coral OR corals) AND (toxicant OR toxicants OR chemical OR chemicals OR biocide OR biocides OR "industrial product" OR "industrial products" OR "consumer product" OR "consumer products" OR "household product" OR "household products" OR "biocidal product" OR "biocidal products" OR disinfect* OR oil OR metal OR metals OR pesticide OR pesticides OR herbicide OR herbicides OR insecticide OR insecticides OR fungicide OR fungicides OR antifoul* OR anti-foul* OR organochlorine OR organochlorines OR "flame retardant" OR "flame retardants" OR detergent OR detergents OR "perfluorinated compound" OR "perfluorinated compounds" OR pharmaceutical OR pharmaceuticals OR "personal care product" OR "personal care products" OR cosmetic OR cosmetics OR PAH OR PAHs OR petroleum OR hydrocarbon OR hydrocarbons OR microplastic OR microplastics OR nanoparticle OR nanoparticles OR nano-particle OR nano-particles OR "endocrine disrupt*" OR "organic compound" OR "organic compounds" OR dispersant OR dispersants OR metalloid OR metalloids OR solvent OR solvents OR petrochemical OR petrochemicals OR additive OR additives OR preservative OR preservatives OR plasticizer OR plasticizers OR hormone OR hormones OR "transformation product" OR "transformation products" OR "degradation product" OR "degradation products" OR byproduct OR byproducts OR by-product OR by-products OR sunscreen OR sunscreens OR "UV filter" OR "UV filters" OR "ultraviolet filter" OR "ultraviolet filters" OR phthalate OR phthalates OR PCB OR PCBs OR cyanide OR cyanides OR chlordecone OR antibiotic OR antibiotics OR nickel OR copper OR zinc OR cadmium OR mercury OR iron) | January 3^rd^ 2022 | 34 |
| Dissertations - **ProQuest Dissertations and Theses** (<https://search.proquest.com/>) (Publicly Available Content Database)  Timespan 2020-2022  Type de source = Thèses et mémoires  ti(coral* AND (toxicant* OR chemical* OR biocide* OR "industrial product*" OR "consumer product*" OR "household product*" OR "biocidal product*" OR disinfect* OR oil OR metal* OR pesticide* OR herbicide* OR insecticide* OR fungicide* OR antifoul* OR anti-foul* OR organochlorine* OR "flame retardant*" OR detergent* OR "perfluorinated compound*" OR pharmaceutical* OR "personal care product*" OR cosmetic* OR PAH* OR petroleum OR hydrocarbon* OR microplastic* OR nanoparticle* OR nano-particle* OR "endocrine disrupt*" OR "organic compound*" OR dispersant* OR metalloid* OR solvent* OR petrochemical* OR additive* OR preservative* OR plasticizer* OR hormone* OR "transformation product*" OR "degradation product*" OR byproduct* OR by-product* OR sunscreen* OR "UV filter*" OR "ultraviolet filter*" OR phthalate* OR PCB* OR cyanide* OR chlordecone OR antibiotic* OR nickel OR copper OR zinc OR cadmium OR mercury OR iron)) | January 3^rd^ 2022 | 0 |
| Dissertations - **OATD Access Theses and Dissertations** (<https://oatd.org/>)  Timespan 2020-2022  title:(coral* AND (toxicant* OR chemical* OR biocide* OR "industrial product*" OR "consumer product*" OR "household product*" OR "biocidal product*" OR disinfect* OR oil OR metal* OR pesticide* OR herbicide* OR insecticide* OR fungicide* OR antifoul* OR anti-foul* OR organochlorine* OR "flame retardant*" OR detergent* OR "perfluorinated compound*" OR pharmaceutical* OR "personal care product*" OR cosmetic* OR PAH* OR petroleum OR hydrocarbon* OR microplastic* OR nanoparticle* OR nano-particle* OR "endocrine disrupt*" OR "organic compound*" OR dispersant* OR metalloid* OR solvent* OR petrochemical* OR additive* OR preservative* OR plasticizer* OR hormone* OR "transformation product*" OR "degradation product*" OR byproduct* OR by-product* OR sunscreen* OR "UV filter*" OR "ultraviolet filter*" OR phthalate* OR PCB* OR cyanide* OR chlordecone OR antibiotic* OR nickel OR copper OR zinc OR cadmium OR mercury OR iron)) | January 3^rd^ 2022 | 12 |
| Dissertations - **French thesis repository** (<https://www.theses.fr/>)  Search on title with keywords “corail” or “coraux”. Timespan 2020-2022 | January 3^rd^ 2022 | 3 |
| Specialist website - **Australian Institute of Marine Science** (<https://www.aims.gov.au/>)  Timespan 2020-2022 | January 4^th^ 2022 | Service unavailable |
| Specialist website - **Coral Health and Monitoring Program** (NOAA, (<https://www.coral.noaa.gov/>) Timespan 2020-2022 | January 4^th^ 2022 | 0 |
| Specialist website - **Coral traits database** (<https://coraltraits.org/>)  Timespan 2020-2022 | January 4^th^ 2022 | 0 |
| Specialist website - **Ecotox knowledge base of the United States Environmental Protection** **Agency** (EPA, <https://cfpub.epa.gov/ecotox/>) Timespan 2020-2022 | January 4^th^ 2022 | 2 |
| Specialist website - **French Coral Reef Initiative** (IFRECOR, <https://ifrecor.fr>)  Timespan 2020-2022 | January 4^th^ 2022 | 1 |
| Specialist website - **French Research Institute for Exploitation of the Sea** (IFREMER, <https://wwz.ifremer.fr/>) Timespan 2020-2022 | January 4^th^ 2022 | 3 |
| Specialist website - **International Coral Reef Initiative** (ICRI, <https://www.icriforum.org/>) Timespan 2020-2022 | January 4^th^ 2022 | 0 |
| Specialist website - **International Coral Reef Society** (ICRS, <http://coralreefs.org/>)  Timespan 2020-2022 | January 4^th^ 2022 | 0 |
| Specialist website - **LabEx CORAIL** (<https://www.labex-corail.fr/>)  Timespan 2020-2022 | January 4^th^ 2022 | 0 |
| Specialist website - **ReefBase - A global information system for coral reefs** (<http://www.reefbase.org/>) Timespan 2020-2022 | January 4^th^ 2022 | 0 |
| Specialist website - **The Endocrine Disruption Exchange (TEDX) List of Potential Endocrine** **Disruptors** (<https://endocrinedisruption.org/interactive-tools/tedx-list-of-potential-endocrine-disruptors>) Timespan 2020-2022 | January 4^th^ 2022 | 0 |
| **TOTAL** | | **2906** |

The two publication databases Scopus and Web of Science Core Collection (WOS CC) were accessed through a CNRS (the French National Centre for Scientific Research) subscription.

We had access to the following WOS CC Citation Indexes: Science Citation Index Expanded (SCI-EXPANDED, 1900-present); Social Sciences Citation Index (SSCI, 1956-present); Arts & Humanities Citation Index (A&HCI, 1975-present); Conference Proceedings Citation Index- Science (CPCI-S, 1998-present); Conference Proceedings Citation Index- Social Science & Humanities (CPCI-SSH, 1998-present); Emerging Sources Citation Index (ESCI, 2015-present); Current Chemical Reactions (CCR-EXPANDED, 1985-present, includes Institut National de la Propriété Industrielle structure data back to 1840); Index Chemicus (IC, 1993-present).
